# Contribution of tRNA sequence and modifications to the decoding preferences of *E. coli* and *M. mycoides* tRNA^Gly^UCC for synonymous glycine codons

**DOI:** 10.1093/nar/gkad1136

**Published:** 2023-12-05

**Authors:** Maria Kompatscher, Karolina Bartosik, Kevin Erharter, Raphael Plangger, Fabian Sebastian Juen, Christoph Kreutz, Ronald Micura, Eric Westhof, Matthias D Erlacher

**Affiliations:** Institute of Genomics and RNomics, Biocenter, Medical University of Innsbruck, Innrain 80-82, 6020 Innsbruck, Austria; Institute of Organic Chemistry, Center for Molecular Biosciences Innsbruck, University of Innsbruck, Innrain 80-82, 6020 Innsbruck, Austria; Institute of Organic Chemistry, Center for Molecular Biosciences Innsbruck, University of Innsbruck, Innrain 80-82, 6020 Innsbruck, Austria; Institute of Organic Chemistry, Center for Molecular Biosciences Innsbruck, University of Innsbruck, Innrain 80-82, 6020 Innsbruck, Austria; Institute of Organic Chemistry, Center for Molecular Biosciences Innsbruck, University of Innsbruck, Innrain 80-82, 6020 Innsbruck, Austria; Institute of Organic Chemistry, Center for Molecular Biosciences Innsbruck, University of Innsbruck, Innrain 80-82, 6020 Innsbruck, Austria; Institute of Organic Chemistry, Center for Molecular Biosciences Innsbruck, University of Innsbruck, Innrain 80-82, 6020 Innsbruck, Austria; Université de Strasbourg, Institut de Biologie Moléculaire et Cellulaire, Architecture et Réactivité de l’ARN, CNRS UPR 9002, 2, allée Konrad Roentgen, F-67084 Strasbourg, France; Institute of Genomics and RNomics, Biocenter, Medical University of Innsbruck, Innrain 80-82, 6020 Innsbruck, Austria

## Abstract

tRNA superwobbling, used by certain bacteria and organelles, is an intriguing decoding concept in which a single tRNA isoacceptor is used to decode all synonymous codons of a four-fold degenerate codon box. While *Escherichia coli* relies on three tRNA^Gly^ isoacceptors to decode the four glycine codons (GGN), *Mycoplasma mycoides* requires only a single tRNA^Gly^. Both organisms express tRNA^Gly^ with the anticodon UCC, which are remarkably similar in sequence but different in their decoding ability. By systematically introducing mutations and altering the number and type of tRNA modifications using chemically synthesized tRNAs, we elucidated the contribution of individual nucleotides and chemical groups to decoding by the *E. coli* and *M. mycoides* tRNA^Gly^. The tRNA sequence was identified as the key factor for superwobbling, revealing the T-arm sequence as a novel pivotal element. In addition, the presence of tRNA modifications, although not essential for providing superwobbling, was shown to delicately fine-tune and balance the decoding of synonymous codons. This emphasizes that the tRNA sequence and its modifications together form an intricate system of high complexity that is indispensable for accurate and efficient decoding.

## Graphical Abstract

**Graphical Abstract:**
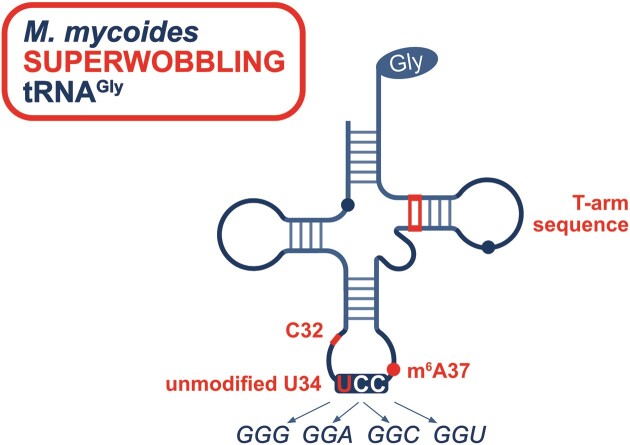

## Introduction

**Figure 1.**
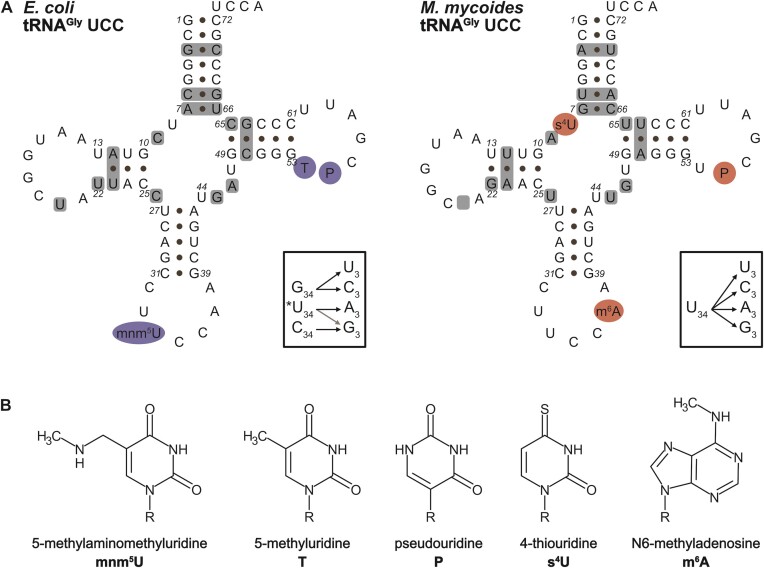
tRNA sequences and modifications. (**A**) Secondary structure of tRNA^Gly^ UCC from *E. coli* and *M. mycoides*. Decoding of the four synonymous glycine codons (GGN) in *E. coli* requires three tRNA^Gly^ isoacceptors with the anticodons CCC, GCC, and *UCC (left box; ‘*’ modified U). In contrast, the *M. mycoides* tRNA^Gly^ with the anticodon UCC is capable of decoding the four GGN codon (right box). Grey shaded boxes in the tRNA structures indicate the sequence differences between the *E. coli* and *M. mycoides* tRNA^Gly^ UCC. The D-arm of the *M. mycoides* tRNA is one nucleotide smaller compared to *E. coli* (indicated by an empty grey box). The modified positions are highlighted in blue (*E. coli*) or red (*M. mycoides*). (**B**) The chemical structures of the indicated tRNA modifications decorating the tRNAs are depicted.

Translation is a central process common to all living organisms. Although the basic concept and mechanism is shared by all known translation systems, numerous differences and variations have evolved (1,2). One particularly intriguing aspect that varies significantly between different organisms is the decoding strategies used to translate the genetic information into protein. While the genetic code is highly conserved across species, the number of tRNA types required to decode all sense codons varies widely. For example, eukaryotes require between 41 and 55 tRNA types (3), while bacteria use 28–46 (4). Mammalian mitochondria have further reduced the number of tRNA types required for translation to a total of 22 (5,6). The reason that different numbers of tRNA types can decode all sense codons lies in the nature of the codon/anticodon interaction. During the decoding process, only the first and second nucleotide of the codon are strictly required to form canonical Watson–Crick (W/C) base pairs with the nucleotides at position 36 and 35 of the tRNA, respectively. This is not the case for the interaction between the third nucleotide of the codon and the tRNA nucleotide at position 34, the first anticodon position. This so-called wobble position (7) tolerates different base pairing geometries, such as G–U wobble base pairs and other non-W/C base pairs (8–10). These interactions are often facilitated or stabilized by the presence of tRNA modifications, making them critical elements for precise and efficient translation. More than 100 different RNA nucleoside derivatives have been identified within tRNAs throughout all kingdoms of life (11). Such modifications can be broadly categorized into body and anticodon loop modifications. Body modifications are primarily thought to contribute to the tRNA structure, folding and stability. Anticodon loop modifications are mainly considered to modulate decoding during translation either directly (via modification at nucleotide 34) or indirectly (via modifications at nucleotides 32, 37, 38) by promoting interactions that enable or stabilize non-W/C base pairs, thereby increasing the selectivity and accuracy of translation (8,10,12–14). This functional variability is reflected in their chemical composition, ranging from simple 2’*O*-methylations, to complex modifications like wybutosine (W) present at nucleotide 37 or 5-carboxymethoxyuridine (cmo^5^U) and derivatives present at nucleotide 34 both requiring multiple steps and modifying enzymes for their biosynthesis (11,15,16). *Escherichia coli* and *Mycoplasma mycoides* are representatives of bacteria that use quite different decoding strategies. The well-studied model organism *E. coli* expresses 41 tRNA types encoded by 87 tRNA genes (17). On the contrary, *M. mycoides*, belonging to the class of *Mollicutes*, which are the smallest and simplest self-replicating bacteria depends on the expression of only 28 different tRNA types encoded by 30 tRNA genes (17–19). In order to compensate for the small number of tRNA types, *Mycoplasma* species as well as mitochondria and chloroplasts employ a remarkable decoding concept (20–22). For example, whereas *E. coli* decodes the four-fold degenerate codon boxes for glycine (GGN), alanine (GCN), valine (GUN), proline (CCN), serine (UCN) and leucine (CUN) by two to three tRNA isoacceptors, *M. mycoides* employs only a single tRNA (17). It has been postulated that for this to occur, these *M. mycoides* tRNAs must either exhibit four-way wobble pairing capability (20) or lack engagement with the third codon position, as suggested by the ‘two-out-of-three’ hypothesis (23). All these tRNAs overcoming the standard rules of decoding (7) by extended wobbling, so-called superwobbling tRNAs, share a common feature, which is an unmodified uridine at position 34 (5,24). No additional common identity element has yet been identified. The four-fold degenerate codon box glycine is one example for the different decoding concepts employed by *E. coli* and *M. mycoides*. In *E. coli*, the four synonymous glycine codons GGA, GGC, GGG and GGU are decoded by three tRNA isoacceptors, namely tRNA^Gly^ CCC, GCC and UCC. In *M. mycoides*, the tRNA^Gly^ UCC is the only tRNA responsible for decoding of all the four glycine codons. The comparison of the tRNA^Gly^ UCC of both bacteria revealed that the sequences are largely shared between the two tRNAs (Figure 1A). In particular, the complete nucleotide sequence within the anticodon stem–loop is identical. However, the modifications in the body and in the anticodon loop are different. The *E. coli* tRNA^Gly^ UCC harbors 5-methylaminomethyluridine (mnm^5^U) at position 34, as well as 5-methyluridine (T) and pseudouridine (P) within the T-loop at positions 54 and 55, respectively (Figure 1). In contrast, the tRNA^Gly^ UCC of *M. mycoides* carries 4-thiouridine (s^4^U) at position 8, N6-methyladenosine (m^6^A) at position 37 and P at position 55 but not the usual T at position 54 (Figure 1). The body modifications s^4^U8, T54 and P55 are primarily considered to stabilize the L-shaped tRNA structure (25,26). The s^4^U8 modification is a commonly present modification in tRNAs, and, interestingly, it is also present in the *E. coli* tRNA^Gly^ CCC and GCC isoacceptors in combination with dihydrouridine (D) within the D-arm (27) (Supplementary Figure S1). Structure analysis of a functional tRNA showed stacking of the 4-thio group on the residue at position 13 and the formation of a W/C or Hoogsteen base pair of s^4^U8 with the nucleotide at position 14 (Supplementary Figure S2) (28). T54 was identified to form an intraloop reverse Hoogsteen base pair with A58 (29) and to stabilize the tRNA structure by increasing the melting temperature of the tRNA (31). P55 is involved in tRNA fold formation (25) due to efficient base stacking ability and base pairing with the G18 within the D-loop (29). mnm^5^U34 and m^6^A37 within the anticodon loop are thought to modulate the decoding capability of the tRNA (32–34). mnm^5^U34 was proposed to stabilize decoding of purine-ending codons, which is especially important for split codon box tRNAs (8,35). m^6^A37, as well as other modified As at position 37, have been proposed to stabilize the codon/anticodon interaction, which is particularly critical in the case of a weak A–U or U–A base pair between nucleotides anticodon first position and codon third position (13), and to contribute to the preorganization of the anticodon loop (34).

Since *M. mycoides* tRNA^Gly^ UCC, in contrast to *E. coli* tRNA^Gly^UCC, can decode all four synonymous codons, we wondered to what extent specific sequence elements or tRNA modifications contribute to the difference in their decoding capacity. Therefore, we established an *in vitro* translation approach based on splinted ligation (Supplementary Figure S3) (36–38). This allowed us to generate differentially modified tRNAs from a set of synthetic RNA oligonucleotides carrying different modifications at different positions. Thus, the contribution of a single modification, but also of a combined set of modifications, on translation can be assessed. We generated tRNA^Gly^ UCC from *E. coli* and *M. mycoides* varying in their sequence and modifications and determined to what extent their decoding capacity was affected. We thoroughly characterized these tRNAs and identified the contribution of different sequence elements and modifications to tRNA superwobbling. This not only broadens our understanding of how modifications and sequence affect the decoding, but also reveals the basis for alternative decoding strategies employed by organisms with reduced genome size.

## Materials and methods

### Plasmids

To generate minimal mRNAs encoding the peptide MRFGRFGEGRFGRF* (1719.98 Da), a short coding sequence (5′ – ATG AGA TTY GGN AGA TTY GGN GAA GGN AGA TTY GGN AGA TTY TAA – 3′; N indicates any of the four canonical bases, and Y indicates pyrimidines), was cloned downstream of the T7 promotor replacing the folA gene of the DHFR control plasmid (NEB). Cloning was performed using the Q5® Site-Directed Mutagenesis Kit (NEB, E0554S) according to manufacturer's protocol. Primers were designed using the NEBaseChanger™ tool and purchased from Integrated DNA Technologies (IDT). Plasmids were harvested and purified using the Monarch® Plasmid Miniprep Kit (NEB, T1010). The sequence of each construct was verified by Sanger Sequencing (Eurofins Genomics, Mix2Seq Kit NightXpress).

The firefly luciferase (fLuc) gene (Supplementary Figure S4) was introduced into the multiple cloning site of a pUC19 plasmid according to (39).

### tRNA transcription

tRNA sequences were obtained from the tRNA Leipzig Database (http://trnadb.bioinf.uni-leipzig.de/) (40). DNA templates for transcription were generated by Phusion Green High-Fidelity DNA Polymerase (F534L, Thermo Scientific™) based on overlapping primers (41) purchased from IDT (Supplementary Table S1). The forward primer harbored a BamHI sequence, T7 promotor, as well as the 5′ part of the respective tRNA sequence. The reverse primer encoded the 3′ part of the tRNA sequence. In order to ensure a defined 3′ end of the DNA template, the two first 5′ nucleotides of the reverse primer were 2*'O*-methylated (42). Transcription by the HiScribe™ T7 High Yield RNA Synthesis Kit (E2040S, NEB) was performed according to manufacturer's protocol for small RNAs. The template was removed by a DNase treatment using 1 U of DNase 1 (Thermo Scientific™, EN0521) for 30 min at 37°C. The RNA was purified using the Monarch® RNA Cleanup Kit (50 μg) (NEB, T2040). The concentration was determined using a photo spectrometer (NanoDrop® ND-1000).

### RNase H mediated selective tRNA depletion of *E. coli* tRNA bulk

Native *E. coli* tRNA bulk included in the PURExpress® Δ(aa, tRNA) Kit (NEB, E6840S) was depleted for up to three tRNA^Gly^ isoacceptors by RNase H. The protocol for the selective depletion of tRNA^Gly^ CCC, GCC and UCC by RNase H digest in presence of DNA antisense oligonucleotides was adapted from (43). For the depletion, 100 μg tRNA bulk were mixed with 400 pmol of antisense DNA (Supplementary Table S2) in 200 mM NaCl and 100 mM Tris–HCl (pH 8.0) in a volume of 60 μl. The solution was denatured for 2 min at 80°C, cooled down to 22°C (0.1°C/s) and incubated for 5 min at 22°C. Subsequently, 10x RNase H buffer (f.c. 1×), DTT (f.c. 10 μM) and 20 U RNase H (NEB, M0297S) were added to a final volume of 80 μl and incubated for 1 h at 37°C in a thermo cycler. Afterwards, the antisense DNA was removed by the addition of RQ1 DNase (Promega, M6101) and incubation for 45 min at 37°C. The amount of DNase was dependent on the number of tRNAs to be depleted (10 U, 15 U or 20 U for one, two or three antisense DNA oligonucleotides, respectively). The RNA was purified using the Monarch® RNA Cleanup Kit (500 μg) (NEB, T2050S). The concentration was determined using photo spectrometry (NanoDrop® ND-1000) and the concentration was adjusted to 10 μg/μl.

### Chemical synthesis of RNA

#### RNA solid-phase synthesis

RNA synthesis was performed on an H6 GeneWorld DNA/RNA automated synthesizer (K&A, Laborgeraete GbR, Germany) on a 1.0 μmol scale or on an ABI 391 PCR-MATE (Applied Biosystems) on a 1.4 μmol scale using standard phosphoramidite chemistry and optimized in-house written synthetic cycles. Standard 2′-O-TOM or 2′-*O*-TBDMS and acetyl protected nucleoside phosphoramidite building blocks (ChemGenes), 2′-*O*-TBS 1000 Å CPG solid support (ChemGenes) and in-house synthesized S4-cyanoethyl-protected 4-thiouridine as well as *N*-trifluoroacetyl (TFA) protected 5-aminomethyluridine (nm^5^U) and 5-methylaminomethyluridine (mnm^5^U) phosphoramidites were used. On the K&A synthesizer, detritylation, coupling, capping and oxidation reagents were dichloroacetic acid/1,2-dichloroethane (4/96), phosphoramidite/acetonitrile (100 mM) and benzylthiotetrazole/acetonitrile (300 mM), Cap A/Cap B (1/1) (Cap A: 4-(dimethylamino)pyridine/acetonitrile (500 mM), Cap B: acetic anhydride/sym-collidine/acetonitrile (2/3/5)) and iodine (20 mM) in tetrahydrofuran/pyridine/H_2_O (35/10/5). On the ABI synthesizer, detritylation and capping solutions were exchanged to 5%-dichloroacetic acid in anhydrous toluene, Cap A: acetic anhydride/lutidine/tetrahydrofuran 1/1/8 (v/v/v) and Cap B: tetrahydrofuran/*N*-methylimidazole 86/14 (v/v), respectively. Solutions of phosphoramidites and tetrazole were dried over activated molecular sieves (3 Å) overnight.

#### Deprotection and purification of RNA

For deprotection of unmodified RNA, the solid support was mixed with aqueous methylamine (40%, 0.50 ml) and aqueous ammonia (28%, 0.50 ml) for 20 min at 65°C. The supernatant was removed and the solid support was washed three times with H_2_O/THF (1.0 ml; 1/1). Combined supernatant and washings were evaporated to dryness and the residue was dissolved either in a solution of tetrabutylammonium fluoride in tetrahydrofuran (1.0 M, 1.5 ml) in the case of 2′-*O*-TOM or in a mixture of 300 μl anhydrous dimethylsulfoxide and 375 μl triethylamine trihydrofluoride for 2′-*O*-TBDMS protecting groups. The solution was subsequently incubated for 16 h at 37°C for the complete removal of the 2′-*O*-silyl protecting groups. Then, the reaction was quenched by addition of triethylammonium acetate/H_2_O (1.0 M, 1.5 ml, pH 7.4). Eventually present tetrahydrofuran was removed under reduced pressure and the sample was desalted by size-exclusion column chromatography (GE Healthcare, HiPrep™ 26/10 Desalting; Sephadex G25) eluting with H_2_O; collected fractions were evaporated and the RNA dissolved in H_2_O (1 ml).

For deprotection of amino modified oligonucleotides, the solid support in the synthesis cartridge was pretreated with 40 ml of 10% diethylamine in anhydrous acetonitrile at a flow rate of 2 ml min^−1^ using two syringes. After washing with 40 ml acetonitrile the solid support was dried *in vacuo* and the deprotection was continued according to the standard procedure described above.

Oligonucleotides modified with 4-thiouridine were treated with 1,8-diazabicyclo[5.4.0]undec-7-en (DBU) in acetonitrile (1.0 M, 2 ml) for 3 h at room temperature. The resin was washed three times with acetonitrile (0.5 ml), dried *in vacuo* and incubated with a mixture of aqueous methylamine (40%, 0.50 ml), aqueous ammonia (28%, 0.50 ml) and dithiothreitol (50 mM) for 20 min at 65°C. The supernatant was removed and the solid support was washed three times with H_2_O/ethanol (1.0 ml; 1/1). Combined supernatant and washings were evaporated to dryness and the residue was dissolved in a mixture of dry DMSO (200 μl) and triethylamine trihydrofluoride (275 μl) and heated for 2.5 h at 65°C. After cooling down to room temperature, sodium acetate (3.0 M, 25 μl) and ethanol (1.0 ml) were added and the RNA was stored at 20°C overnight. After centrifugation (13 000 rpm, 20 min, 4°C), the supernatant was discarded and the pellet was washed twice with ethanol (0.5 ml). The pellet was then dried *in vacuo* and dissolved in H_2_O (1 ml).

The crude RNA was purified by anion exchange chromatography (Thermo Scientific Ultimate 3000 HPLC System) on a semipreparative Dionex DNAPac® PA-100 column (9 mm × 250 mm) at 80°C with a flow rate of 2 ml/min (eluent A: 20 mM NaClO_4_, 25 mM Tris·HCl, pH 8.0, 20 v/v % acetonitrile; eluent B: 600 mM NaClO_4_, 25 mM Tris·HCl, pH 8.0, 20% v/v acetonitrile). Fractions containing RNA were evaporated and the residue redissolved in 0.1 M triethylammonium bicarbonate solution (10–20 ml), loaded on a C18 SepPak Plus® cartridge (Waters/Millipore), washed with H_2_O, and then eluted with acetonitrile/H_2_O (1/1). Crude and purified RNA were analyzed by anion exchange chromatography (Thermo Scientific Ultimate 3000 HPLC System) on a Dionex DNAPac® PA-100 column (4 mm x 250 mm) at 80°C with a flow rate of 1 ml/min. For RNA up to 15 nucleotides in length, a gradient of 0–40% B in 30 min was applied; for longer RNA a gradient of 0–60% B was applied; eluent A: 20 mM NaClO_4_, 25 mM Tris·HCl, pH 8.0, 20% v/v acetonitrile; eluent B: 600 mM NaClO_4_, 25 mM Tris·HCl, pH 8.0, 20% v/v acetonitrile. HPLC traces were recorded at UV absorption by 260 nm.

The synthesis yield was determined via UV-photometrical analysis on an Implen NanoPhotometer 7122 V2.0.0 and the mass was verified using ion pair reversed phase LC-ESI-MS analysis on a Finnigan LCQ Advantage Max mass spectrometer equipped with an electrospray ionization (ESI) interface coupled to a Thermo Scientific Dionex Ultimate 3000 HPLC system. The sample was applied to a Thermo Scientific DNAPacTM RP column (2.1 × 50 mm) and eluted using an increasing gradient from Buffer A: 8.6 mM triethylamine 100 mM hexafluoriisopropanol in ddH_2_O (miliQ) to Buffer B: MeOH.

### Splinted RNA ligation

Specifically modified tRNAs were generated via splinted ligation of up to three synthetic RNA oligonucleotides (Supplementary Table S3) using reverse complement DNA splinter (Supplementary Table S2). The enzymatic ligation protocol was adapted from (37,44). For one ligation usually 1 nmol of each RNA fragment and the respective DNA splint were combined in a final volume of 70 μl and denatured at 90°C for 2 min. Subsequently, the solution was passively cooled down to room temperature over 15 min. For the ligation reaction 10 μl 10× T4 DNA ligase buffer, 10 μl 50% PEG4000, and 50 U T4 DNA ligase (Thermo Scientific) were added and incubated for 3 h at 37°C. RQ1 DNase (Promega, M6101) (5 U/reaction) was added and incubated 30 min at 37°C. The ligation was denatured for 2 min at 95°C containing 0.5× volume formamide before loading on a denaturing 10% polyacrylamide gel (29:1; 7 M urea). The ligated product was detected by UV-shadowing. The respective band was cut out and fragmented into small pieces and subsequently collected in a fresh Eppendorf tube. The sample was overlaid with RNA gel elution buffer containing 300 mM NaCl, 0.2% SDS, 60 mM NaOAc (pH 5.2) according to (45). The sample was shaken overnight at 4°C following an incubation at 60°C for 1 h. The samples were cooled on ice before purification using the Monarch® RNA Cleanup Kit (10 μg) (NEB, T2030L). The concentration was determined using photo spectrometry (NanoDrop® ND-1000).

As an alternative to polyacrylamide gel electrophoresis, analysis and purification of the ligation products were performed by anion-exchange HPLC (for conditions see section *‘Deprotection and purification of RNA’*). After incubation at 37°C for 3 h, the ligation reaction was extracted twice with phenol/chloroform/isoamyl alcohol solution (25/24/1 v/v/v) and then twice with chloroform to remove any remaining phenol from the mixture. The solution was concentrated *in vacuo* and purified by AE-HPLC on an analytical column. The ligated products were obtained with high purity, that was confirmed by AE-HPLC analysis.

### *In vitro* translation

All *in vitro* translations were performed using the PURExpress® Δ(aa, tRNA) Kit (NEB, E6840S). For *in vitro* translation 10 pmol tRNA^fMet^ CAU, 30 pmol tRNA^Phe^ GAA, 30 pmol tRNA^Arg^ UCU transcripts and 15 pmol of the transcribed or modified tRNA^Gly^ were prepared in a PCR tube and lyophilized in a Concentrator Plus (Eppendorf). Due to a specific contamination of the PURExpress® Δ(aa, tRNA) Kit with native tRNA^Glu^, the addition of an *in vitro* transcribed tRNA^Glu^ was dispensable. Lyophilized tRNAs were dissolved in the reaction mixture containing 50 ng of plasmid template, 1.25 μl Solution A, 1.875 μl Solution B, 0.63 μl amino acid mix, 0.5 μl [^35^S]-Met/Cys (Hartmann Analytic; 10 mCi/ml) in a final volume of 6.25 μl. The reactions were incubated for 2 h at 37°C. The translation was stopped on ice by adding 2 μl of 4× Laemmli buffer (8% SDS, 40% glycerol, 350 mM Tris pH 8.8, 400 mM β-mercaptoethanol, 0.1% bromophenol blue). The peptide yields were determined by a Tricine-SDS-PAGE with a 16% separating (6 M urea), a 10% spacing and 4% stacking gel (46). Afterwards, the gel was shaken for approximately 5 min each in staining (50% ethanol, 5% acetic acid) and destaining (20% ethanol, 7.5% acetic acid) solution. The gel was dried on a Whatman paper for 1 h at 70°C in a slab gel dryer (Cleaver Scientific), exposed to a phosphor screen overnight and scanned by Typhoon™ FLA 9500 (GE Healthcare). The bands were quantified using the ImageQuantTL software.

For the luciferase assays, 15 pmol tRNA transcript or synthetic tRNA^Gly^ isoacceptors were lyophilized in PCR tubes in a Concentrator Plus (Eppendorf). When all *E. coli* tRNA^Gly^ isoacceptors were employed simulaneously, 7.5 pmol, 3.25 pmol and 3.25 pmol tRNA^Gly^ GCC, CCC and UCC transcripts were used, respectively. 10.7 μg tRNA bulk or depleted tRNA bulk were added together with 36 ng DNA template, 0.9 μl Solution A, 1.36 μl Solution B, and 0.45 μl amino acid mix. The reactions containing a total volume of 4.5 μl were incubated for 2 h at 37°C. To determine the luciferase activity 25 μl of the luciferase assay reagent (Promega, E1483) was prepared in a clear bottom, black 96 well plate (Greiner Bio-one). 2 μl of the translation reaction was pipetted to the wall of the well and all reactions were started simultaneously by short centrifugation at 1000×g. The luminescence of two technical replicates per sample was measured in a BMG Labtech FLUOstar Omega plate reader with a detection time of 9.5 s and a gain of 3000.

### Statistical analysis

The quantitative data from the *in vitro* translation assays were subjected to a two-step statistical analysis using R. First, an ANOVA comparison was performed. In case the ANOVA model was significant (*P*-value < 0.05), the statistical analysis was continued with Dunnett's post-hoc test. Dunnett's test was used to determine which normalized sample means were significantly different from the reference sample mean. For fLuc assays, the ‘3× depletion’ shown in the graphs served as a quality control sample and was therefore not included in the statistical analysis.

## Results

tRNA^Gly^ UCC from *M. mycoides* and *E. coli* (Figure 1) are very similar in their sequence differing only in some nucleotides within the tRNA body. The sequence alignments (Supplementary Figure S1) revealed an identical anticodon stem-loop (nucleotide 27 to 43), while the acceptor stem is GC-rich in *E. coli* compared to *M. mycoides*, in which two of the first six G–C pairs from *E. coli* are changed into A-U pairs in *M. mycoides*. However, there is one A–U pair in *E. coli* which is replaced by a G–C pair in *M. mycoides* located at the end of the acceptor stem (position 7–66). The sequences of the T-loops are identical, but the T-stem contains only G–C in *E. coli* while it contains a G-U and A-U pair at the beginning of the T-stem in *M. mycoides*. More variations are found in the D-arm. The D-loop is one nucleotide smaller in *M. mycoides* but otherwise the sequences are identical. Further, there is a rare C9 in *E. coli* while *M. mycoides* harbors the more common A9. In the common tertiary structure of tRNAs, the base pair 12-23 makes a triple interaction with residue 9 (26,47,48) leading probably to A9/A23 non-W/C base pair in *M. mycoides* containing the base triplet A9, U12–A23. In bacterial sequences, all tRNA^Gly^ with C9 have an A12–U23 pair (17) of which the potential contact between C9 and U23 is not known and needs to be verified. An additional difference in the D-stem is the U13/U22 in *E. coli* replaced by a U13–G22 in *M. mycoides*. In the variable loop, *E. coli* harbors a GA while *M. mycoides* a UG sequence. To identify the sequence elements and the modifications responsible for this difference in the decoding capability of these tRNAs, we generated tRNA^Gly^ variants that differ in their sequence and modifications.

### Decoding of synonymous glycine codons by *E. coli* and *M. mycoides* tRNA^Gly^ isoacceptors

Previous studies have shown that the tRNA^Gly^ UCC of *M. mycoides* is compatible with the *E. coli* translation machinery (49,50). This allows to test the decoding strategy of superwobbling tRNAs of *Mycoplasma* in well-established *E. coli* translation systems. In order to avoid the tRNAs to be modified by *E. coli* enyzmes (51,52), we employed a defined recombinant *in vitro* translation system that lacks all modifying enzymes (53). The first question addressed was whether an *in vitro* transcribed and thus unmodified *M. mycoides* tRNA^Gly^ UCC is still able to decode all four synonymous glycine codons or whether the modified nucleotides are a prerequisite for superwobbling. In addition, the decoding capacity of the unmodified *E. coli* tRNA was determined, since it contains the same anticodon stem-loop sequence as the *M. mycoides* tRNA and an unmodified U34, which is a characteristic feature for extended wobbling.

**Figure 2.**
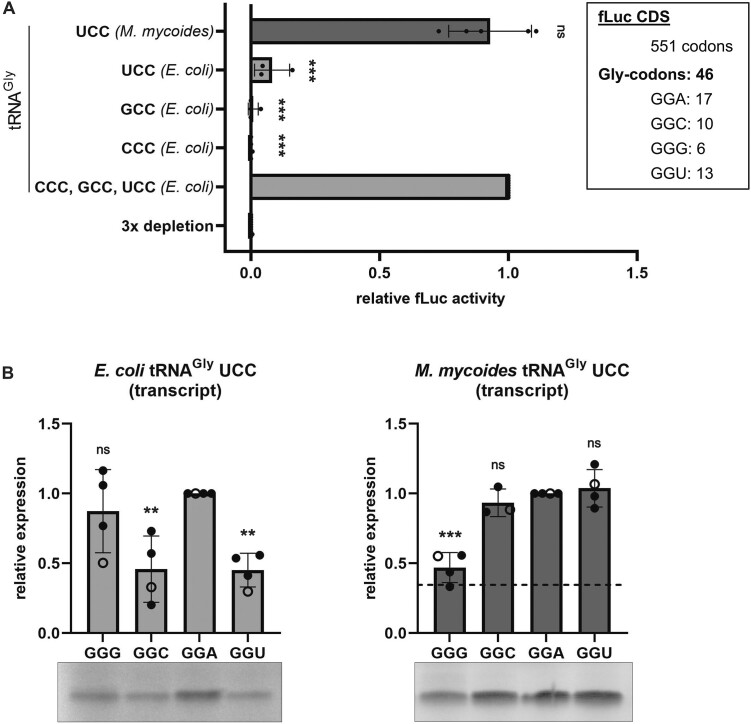
Decoding of synonymous glycine codons by unmodified tRNA^Gly^ isoacceptors. (**A**) *In vitro* translation of the firefly luciferase (fLuc) gene with *E. coli* tRNA bulk depleted in the tRNA^Gly^ isoacceptors was rescued with tRNA^Gly^ isoacceptor transcripts from *E. coli* (light grey) and *M. mycoides* (dark grey). The glycine codon composition of the fLuc coding sequence (CDS) is shown in the upper right (box). Datapoints represent technical duplicates of individual experiments and fLuc activity was normalized to the sample using all three *E. coli* tRNA^Gly^ isoacceptor transcripts. (**B**) Decoding of specific glycine codons by the *E. coli* and *M. mycoides* tRNA transcripts within a short mRNA. Each datapoint represents the quantification of [^35^S]-Met labelled peptides on autoradiographs. Open circles indicate quantifications from the representative gels. Peptide yields resulting from the sequence containing GGA codons were used as reference. The dashed line indicates the peptide yield resulting from translation of the GGA harbouring mRNA by *E. coli* tRNA^Gly^UCC transcript. (**A**, **B**) Mean of individual datapoints with standard deviation is shown. Significance was tested by ANOVA ((**A**) < 2e-16; (**B**) 0.00337 (left); 1.85e-05 (right)) followed by Dunnett's test (0.001 ‘***’, 0.01 ‘**’, 0.05 ‘*’, >0.05 ‘ns’).

To determine the decoding ability of these transcribed tRNAs, the native *E. coli* tRNA^Gly^ isoacceptors within tRNA bulk from *E. coli* were substituted with the tRNAs of interest and tested in *in vitro* translation of the fLuc reporter mRNA containing all four synonymous glycine codons in multiple copies (Figure 2A, Supplementary Figure S4). Therefore, the native *E. coli* tRNA pool was depleted of the three tRNA^Gly^ isoacceptors using RNase H (Supplementary Figure S5A). Long antisense DNA oligonucleotides (Supplementary Table S2) were hybridized to the *E. coli* tRNA^Gly^ isoacceptors and subsequently digested with RNase H, which is specific for RNA-DNA duplexes. Due to the high abundance of tRNA^Gly^ GCC compared to the other two isoacceptors (four gene copies instead of one) (54,55), a single depletion step targeting all three isoacceptors simultaneously was not sufficient. Therefore, a second depletion targeting tRNA^Gly^ GCC was required to further deplete residual amounts of native tRNA (Supplementary Figure S5B). This pool of tRNAs lacking all three tRNA^Gly^ isoacceptors, was subsequently used in the PURExpress Δ(aa, tRNA) translation kit in combination with a fLuc assay. This recombinant PURExpress system was designed to allow the separate addition of tRNAs and amino acids and is therefore an ideal system for testing tRNA pools of different compositions (53,56). The RNase H treated tRNA pool did not provide any luciferase activity, indicating that the tRNA^Gly^ isoacceptors were successfully eliminated. However, translation could be efficiently rescued by the simultaneous addition of the three *in vitro* transcribed *E. coli* tRNA^Gly^ isoacceptors with the anticodons GCC, CCC and UCC. Interestingly, the addition of *E. coli* tRNA^Gly^ UCC alone provided only small amounts of luciferase signal. In contrast, the *M. mycoides* tRNA^Gly^ UCC resulted in comparable luciferase activities as the three *E. coli* tRNA^Gly^ isoacceptors combined, revealing its superwobbling capability even in the absence of all its tRNA modifications (Figure 2A).

To understand how the tRNA^Gly^ UCC from *E. coli* and *M. mycoides* differ in their ability to decode the synonymous glycine codons, we tested each glycine codon individually. Therefore, a defined translation assay employing a minimal mRNA encoding for a 14 amino acid long peptide was established. To determine the decoding capability of the tRNAs for individual synonymous glycine codons, four variations of the coding sequences were generated, differing only in the third nucleotide of the glycine codons (Supplementary Figure S6). Thereby, we were able to observe differences in the efficiency of each tRNA to decode the individual synonymous codons (Figure 2B), which cannot be determined by using the fLuc assay as the coding sequence contains all four codons. Whereas, the unmodified *E. coli* tRNA^Gly^ UCC was most efficient in decoding GGA and GGG codons, the *M. mycoides* tRNA showed lowest decoding efficiency for GGG codons. However, the *M. mycoides* tRNA generally provided higher protein yields than the *E. coli* tRNA^Gly^ UCC, even when decoding the mRNA harboring the least favored GGG codons.

### Contribution of tRNA sequence to superwobbling

**Figure 3.**
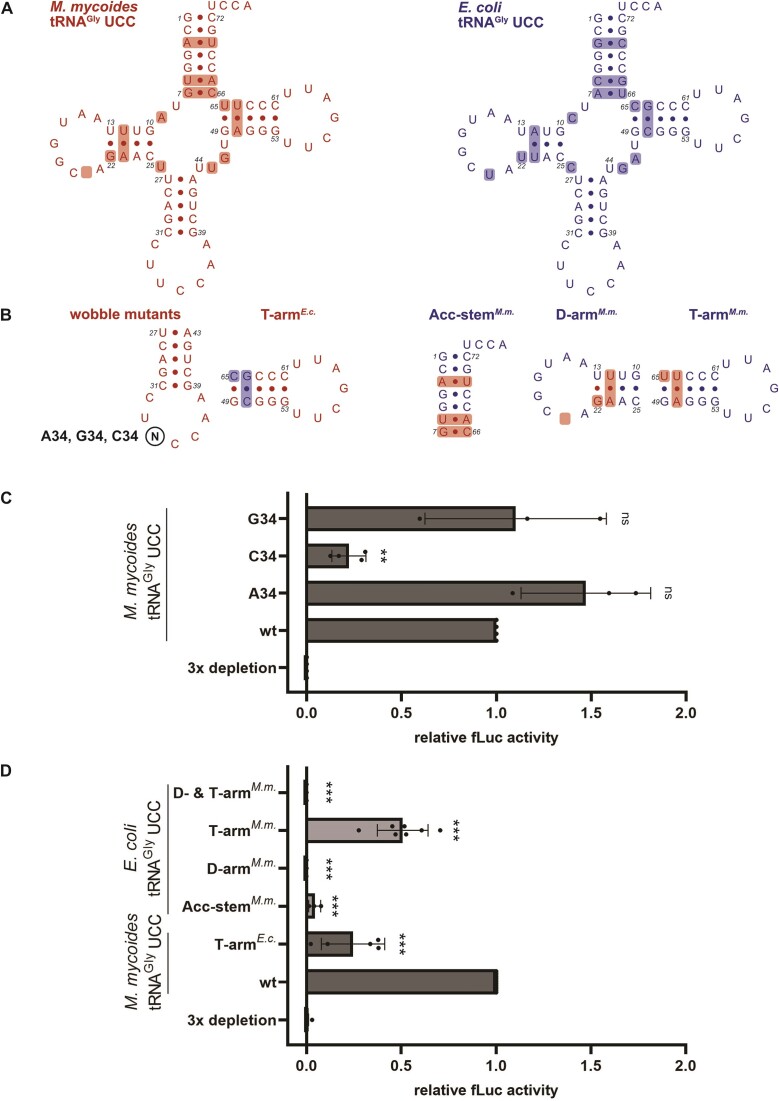
Impact of tRNA sequence on decoding of synonymous glycine codons. (**A**) The secondary structure of the unmodified wild-type (wt) *M. mycoides* (*M.m*.; red) and *E. coli* (*E.c*.; blue) tRNA^Gly^ UCC sequences are shown, with sequence differences highlighted. (**B**) Single nucleotides (wobble mutants) or sequence elements (acceptor (Acc)-stem, D-arm, T-arm) were exchanged between the *M. mycoides* and the *E. coli* tRNA^Gly^. (**C**) *M. mycoides* tRNA mutated at position 34 and (**D**) other *E. coli* and *M. mycoides* tRNA mutants were tested for their ability to sustain fLuc translation in absence of the native *E. coli* tRNA isoacceptors. Datapoints represent technical duplicates of individual experiments. The fLuc activity was normalized to the wt *M. mycoides* tRNA sample. (**C**, **D**) Mean of individual datapoints with standard deviation is shown. Significance was tested by ANOVA ((**C**) 0.000675; (**D**) <2e-16) followed by Dunnett's test (0.001 ‘***’, 0.01 ‘**’, 0.05 ‘*’, >0.05 ‘ns’).

The observation that the *in vitro* transcribed *M. mycoides* tRNA^Gly^ UCC lacking all tRNA modifications, was able to compensate for the loss of all three *E. coli* tRNA^Gly^ isoacceptors suggests that its superwobbling ability is not dependent on the modifications and might be mainly based on the tRNA sequence (Figure 1). To address which nucleotides or sequence elements enable superwobbling, several tRNA mutants of *E. coli* and *M. mycoides* were created (Figure 3A, B).

As the unmodified U34 is the only common feature between all superwobbling tRNAs of mitochondria, chloroplasts and *Mycoplasma* species, we determined the importance of this U34 on decoding of the synonymous glycine codons. We mutated the *M. mycoides* tRNA^Gly^ UCC at the wobble position resulting in tRNAs carrying an ACC, CCC or GCC anticodon and tested for their aminoacylation (Supplementary Figure S7A). Remarkably, the ACC and GCC anticodon carrying tRNAs were able to efficiently compensate for the depletion of the three tRNA^Gly^ isoacceptors, whereas the C34 mutant could only partially sustain translation (Figure 3C). This suggests that U34 is not strictly required for superwobbling in the context of the *M. mycoides* tRNA^Gly^ UCC sequence, nor does it seem to be the sole determinant, as the unmodified U34 within the *E. coli* sequence did not increase the decoding capacity.

Since the anticodon stem-loop sequence of *E. coli* and *M. mycoides* tRNA^Gly^ UCC are identical, the contribution of the T-arm, D-arm and the acceptor stem were determined. Therefore, the respective sequence elements from the *M. mycoides* tRNA were introduced into the *E. coli* tRNA^Gly^ UCC (Figure 3A, B). After efficient aminoacylation of the mutants by the *E. coli* glycyl-tRNA synthetase (GlyRS) has been confirmed (Supplementary Figure S7B), they were tested for their potential to compensate for the depletion of the three *E. coli* tRNA^Gly^ isoacceptors during translation. Indeed, the introduction of the *M. mycoides* T-arm, consisting of the mutation C65U and the substitution of the base pair C50-G64 for A50-U64, resulted in a partial rescue of translation by the mutant *E. coli* tRNA. The exchange of the acceptor stem or the D-arm did not equip the *E. coli* tRNA with the ability to compensate for the loss of the tRNA^Gly^ isoacceptors. Also, the combined introduction of the T- and D-arm did not enable translation of the luciferase mRNA (Figure 3D). Interestingly, even the capability to decode the cognate GGA codon was reduced by the D-arm muntant and completely abolished by the mutant with combined D- and T-arm (Supplementary Figure S7C). Since the T-arm sequence of *M. mycoides* was a key element to improve the superwobbling ability of the *E. coli* tRNA, we wondered how the T-arm sequence of *E. coli* would affect the *M. mycoides* tRNA. Indeed, a decreased luciferase activity was detected (Figure 3D), underlining the importance of the T-arm for the extended decoding capability of the *M. mycoides* tRNA.

### Contribution of tRNA modifications in *E. coli* tRNA^Gly^ UCC to decoding

tRNAs are the most abundantly modified RNA molecules and the importance of these modifications is undisputable. Although, tRNA^Gly^ isoacceptors are in general only sparsely modified in bacteria (27,40), their modifications are still considered to have indispensable functions for tRNA structure and efficient decoding. Especially, modifications at the wobble position are postulated to modulate decoding efficiency and accuracy of the respective tRNAs. The *E. coli* tRNA^Gly^ UCC harbors three modifications (Figure 1): T54 and P55 within the T-arm and the wobble modification mnm^5^U34. This wobble modification was postulated to improve base pairing to G at the third codon position (57,58).

**Figure 4.**
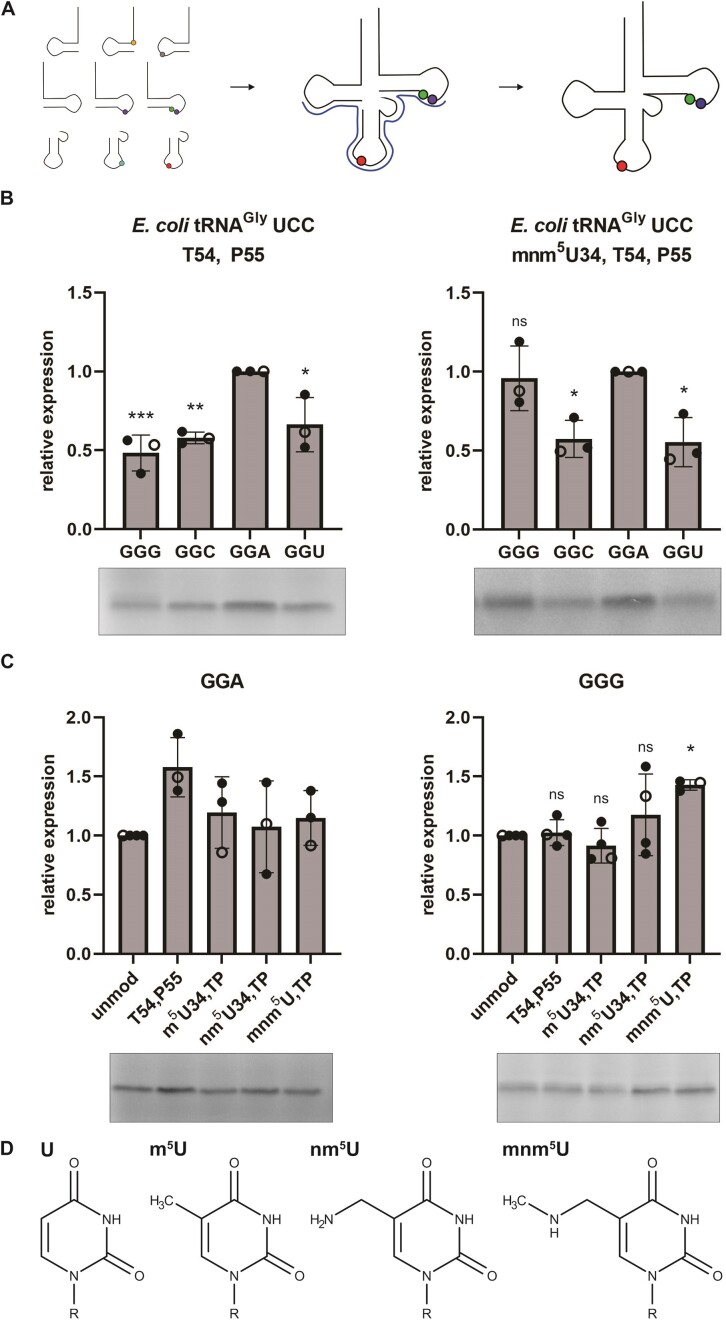
The effect of tRNA modifications on decoding by *E. coli* tRNA^Gly^ UCC. (**A**) Schematic depiction of splinted ligation of differently modified chemically synthesized RNA oligonucleotides to generate full-length site-specifically modified tRNAs. (**B**) Decoding efficiency of the four synonymous glycine codons within a small mRNA by partly or fully modified *E. coli* tRNA^Gly^ UCC. [^35^S]-Met labelled peptides were quantified and normalized to the peptide yields resulting from the GGA containing mRNA. (**C**) Translation efficiency of tRNAs harboring different modifications depicted in (**D**) at position 34. Small mRNAs either containing GGA or GGG codons were *in vitro* translated in presence of [^35^S]-Met and the respective modified *E. coli* tRNA^Gly^ UCC. Peptide yields were normalized to the sample of unmodified tRNA^Gly^ UCC. Open circles indicate datapoints shown in the representative gels. (**B**, **C**) Mean of individual datapoints with standard deviation is shown. Significance was tested by ANOVA ((**B**) 0.00158 (left); 0.00677 (right); (**C**) not significant (left); 0.0204 (right) followed by Dunnett's test (0.001 ‘***’ 0.01 ‘**’ 0.05 ‘*’ >0.05 ‘ns’).

To systematically determine the impact of the modifications on decoding, we generated site-specifically modified tRNAs independent of any modifying enzyme. Thereby, chemically synthesized RNA oligonucleotides were ligated via splinted ligation to result in full-length modified tRNAs (Figure 4A). This approach enabled the site-specific and quantitative introduction of specific modifications or combinations thereof into tRNAs. As first step, only the conserved T-arm modifications T54 and P55 were inserted but U34 was left unmodified. Already the presence of the T-arm modifications significantly modulated decoding of glycine codons (Figure 4B). Whereas the unmodified tRNAs decoded the mRNAs harboring GGA and GGG codons similarly efficient (Figure 2B), the T-arm modifications seemed to be disadvantageous for GGG decoding (Figure 4B). By adding mnm^5^U at position 34, resulting in a fully-modified tRNA^Gly^, the decoding of GGG was significantly improved again, consistent with its proposed role for enhancing the decoding of G-ending codons (9,58) (Figure 4B). These results indicate that the full set of modifications is needed to provide balanced decoding of GGA and GGG, revealing a significant crosstalk between the body and anticodon modifications.

The chemical assembly used here to introduce and to test modifications not only allows the introduction of natural modifications but also other non-standard RNA derivative. Next, we tried to reveal whether the complete mnm^5^U modification is needed for modulating decoding. Therefore, the metabolic precursor nm^5^U and also m^5^U (T) (Figure 4D) were inserted at the wobble position of the *E. coli* tRNA^Gly^ UCC harboring the T-arm modifications. Remarkably, all tested modifications reduced the efficiency of decoding mRNAs harboring GGA glycine codons. Already the presence of a methylation slightly reduced peptide yields. In respect to the GGG codon, only mnm^5^U was able to improve its decoding, whereas the precursor nm^5^U or m^5^U did not change the translation activity (Figure 4C).

### Contribution of tRNA modifications in *M. mycoides* tRNA^Gly^ UCC to decoding

**Figure 5.**
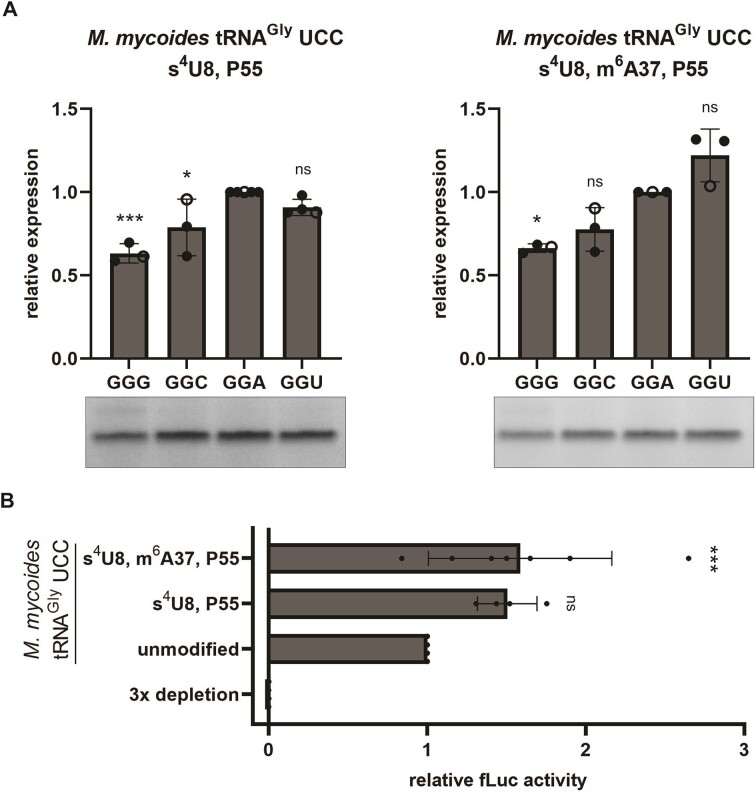
The effect of tRNA modifications on decoding by *M. mycoides* tRNA^Gly^ UCC. (**A**) The *M. mycoides* tRNA^Gly^ UCC carrying different sets of modifications were tested for their capability to decode the synonymous glycine codons. [^35^S]-Met labelled peptides were quantified and normalized to the peptide yields resulting from the GGA containing mRNA. Open circles indicate datapoints shown in the representative gels. (**B**) Depletion of the three *E. coli* tRNA^Gly^ isoacceptors within *E. coli* tRNA bulk was rescued with the differently modified *M. myocides* tRNA^Gly^. The fLuc activities were normalized to the unmodified tRNA sample. (**A**, **B**) Mean of individual datapoints with standard deviation is shown. Significance was tested by ANOVA ((**A**) 0.000424 (left); 0.000782 (right); (**B**) 0.000722) followed by Dunnett's test (0.001 ‘***’, 0.01 ‘**’, 0.05 ‘*’, >0.05 ‘ns’).

Like the *E. coli* tRNA also the *M. mycoides* tRNA^Gly^ UCC carries only a small number of tRNA modifications (Figure 1). To assess the impact of the tRNA body modifications, s^4^U at position 8 and P at position 55 were introduced into the *M. mycoides* tRNA. This partially modified tRNA showed comparable decoding as the unmodified tRNA transcript (Figures 2B and 5A). The additional introduction of the m^6^A modification at position 37 resulted in an enhanced translation of the GGU containing mRNA (Figure 5A).

In addition to translation of the short mRNAs, we employed the luciferase reporter gene assay to determine the ability of the differently modified tRNAs to decode all glycine codons within the luciferase reporter mRNA. Therefore, tRNAs without modifications, tRNAs harboring only the body modifications s^4^U8 and P55, and tRNAs with the complete set of modifications were compared in their potential to provide translation in absence of the native *E. coli* tRNA^Gly^ isoacceptors. Thereby, we observed that the body modifications lead to increased luciferase activities, which was not further improved by m^6^A at position 37 (Figure 5B). This finding indicates that the modifications are not essential to provide the ability to decode all four synonymous glycine codons, but they modulate the efficiency by potentially stabilizing and prestructuring the tRNA in a native fold for an overall better translation performance.

### m^6^A at position 37 enables superwobbling of *E. coli* tRNA^Gly^ UCC

**Figure 6.**
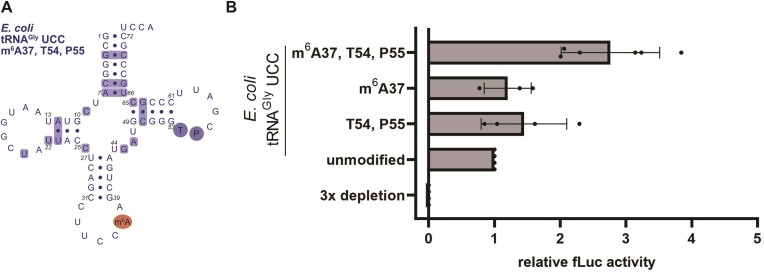
The effect of m^6^A37 on decoding. (**A**) Secondary structure of the hybrid modified *E. coli* tRNA^Gly^ UCC carrying the native T-arm modifications T54 and P55 (shaded in blue) and the *M. mycoides* tRNA modification m^6^A at position 37 (shaded in red). (**B**) Depletion of the three *E. coli* tRNA^Gly^ isoacceptors within *E. coli* tRNA bulk was rescued with the differently modified *E. coli* tRNA^Gly^ UCC. Datapoints represent measurements of technical duplicates of individual experiments. The fLuc activity was normalized to the unmodified tRNA sample. Mean of individual datapoints with standard deviation is shown. Significance was tested by ANOVA (not significant).

Although *M. mycoides* tRNA was highly efficient in decoding all four glycine codons even in absence of its modifications, the presence of them was still beneficial. All superwobbling tRNAs from *M. capricolum*, a *Mollicutes* closely related to *M. mycoides*, of which the sequences and modifications of the complete set of tRNAs are known, harbor either m^6^A or m^1^G at position 37 (18,24). As m^6^A at position 37 was postulated to significantly impact decoding through a structural preorganization of the anticodon loop (34), this modification might contribute to the superwobbling capability of a tRNA. Therefore, m^6^A was inserted in *E. coli* tRNA^Gly^ UCC in combination with the conserved *E. coli* T-arm modifications (Figure 6A) and tested for its ability to compensate for the loss of the *E. coli* tRNA^Gly^ isoacceptors. As controls also a tRNA carrying only the T-arm modifications or only the m^6^A modification were tested alongside in the luciferase reporter assay. Remarkably, m^6^A did provide the *E. coli* tRNA with improved ability to decode all four glycine codons, but interestingly only in combination with the T-arm modifications, T54 and P55 (Figure 6B). This observation suggests the importance of tRNA modifications for structural integrity and preorganization of the anticodon loop to enable efficient protein synthesis.

## Discussion

tRNAs play a crucial role during protein synthesis by enabling decoding of the codons within mRNAs and the integration of the corresponding amino acid into the nascent peptide chains. Different sequence elements and modification patterns have evolved to allow the multiple steps that tRNAs go through in order to provide the ribosome with an adequate supply of amino acids (59,60). To be delivered to the A-site, the tRNA needs to undergo aminoacylation and subsequently form a ternary complex with the GTPase EF-Tu and GTP. At the ribosomal A-site, the anticodon of the tRNA forms an interaction with the mRNA codon. Following decoding, the aminoacyl-tRNA fully accommodates into the A-site, enabling peptide bond formation and translocation to the P-site (61,62). This path of a tRNA during translation, is conserved in all organisms. However, tRNAs vary in their sequence, modifications, and decoding concepts. Remarkable decoding strategies based on superwobbling tRNAs decoding four-fold degenerate codon boxes, have been evolved by certain bacteria and organelles. This resulted in a significantly reduced number of tRNAs required to decode all sense codons (18). To better understand the requirements for extended wobbling, we compared the superwobbling tRNA^Gly^ UCC from *M. mycoides* with the tRNA^Gly^ UCC from *E. coli* using a novel approach based on the ligation of chemically synthesized RNA oligonucleotides.

We generated and tested unmodified and variations of modified *M. mycoides* and *E. coli* tRNA^Gly^UCC for their ability to decode all four synonymous glycine codons. The results show that the modifications of the *M. mycoides* tRNA^Gly^ are not essential to provide superwobbling, which led to the conclusion that its tRNA sequence is the primary prerequisite for extended wobbling (Figure 2). The unmodified U34 and C32, within the *M. mycoides* tRNA^Gly^, were previously identified as crucial elements for its superwobbling ability (49,51,63). However, U34 was not essential for superwobbling by *M. mycoides* tRNA^Gly^. Mutants with A34 or G34 were both highly efficient in decoding all four synonymous glycine codons (Figure 3C). G34 is commonly used in bacterial tRNAs and a loss of decoding specificity has not been described yet (13,40,64). In contrast, A34 is not found in any of the tRNAs except for tRNA^Arg^, where A34 undergoes A-to-I editing (64,65) and A34 has already been shown to base pair with all four nucleotides (52). However, at position 32 the mutation of C to U was detrimental to superwobbling (Supplementary Figure S8) (49,51). The presence of U32 allows the formation of a base pair with A38, thereby affecting the preorganization of the tRNA anticodon loop (49,51,66). The interaction between the nucleotides at position 32 and 38 has been identified to influence the binding affinity to the ribosomal A-site and to balance the different strengths of codon/anticodon interactions to achieve uniform binding (67–71). Since the sequence of the anticodon stem-loop of *M. mycoides* and *E. coli* is identical, it was unexpected that the unmodified *E. coli* tRNA^Gly^ UCC was not able to efficiently decode the four synonymous glycine codons (Figure 2). Only the additional insertion of the *M. mycoides* T-arm into the *E. coli* tRNA extended the decoding capability and thereby revealed an additional sequence element important for superwobbling (Figure 3D). The T-stem sequence has been shown to determine the binding affinity to EF-Tu in accordance with the thermodynamic contributions of the tRNA body and its esterified amino acid (72,73), potentially supporting extended decoding. However, tRNAs from different bacteria within an anticodon class have similar predicted mean Δ*G*° values for EF-Tu binding despite sequence variations in their T-stems (74), arguing against a major impact of EF-Tu.

An alternative explanation could be that the T-arm sequence modulates the structure or folding of the *M. mycoides* and *E. coli* tRNA. The tRNA sequence is primarily responsible for folding into a native and functional tRNA. However, based on the sequence, the partition function will exhibit varied populations, with some sequences adopting numerous folding states while others having a limited number. Among those states, the proportion of native folds is critical for function. Different folding preferences between the tRNAs were calculated from secondary structure predictions, resulting in the correct secondary cloverleaf structure for *M. mycoides* tRNA, but not for *E. coli* (Supplementary Figure S9) (75). UV melting analyses had found application for tRNAs already before (76–79) and can provide an insight into tRNA folding. Indeed, these experiments show differences between the two tRNAs in absence and in presence of Mg^2+^ ions implicating different folding (Supplementary Figure S10). As an alternative approach, NMR melting curves were performed which, in agreement with UV melting, show a higher stability (but not necessarily of the native folding state) of the *E. coli* tRNA compared to the *M. mycoides* tRNA (Supplementary Figure S11). However, due to the complexity of the tRNA structure, the melting curves obtained did not allow conclusions to be drawn about specific structural differences.

Folding of tRNAs is assisted by the presence of modifications. By introducing modifications separately into the tRNAs, the individual contribution of the body and anticodon loop modifications to decoding could be determined. Thereby, we observed a crosstalk between the modifications as well as a modulatory effect on the decoding efficiency of the tRNAs for specific codons. Decoding of GGA, GGC and GGU by the *E. coli* tRNA^Gly^ UCC (Figure 4) was enhanced by the introduction of the T-arm modifications T54 and P55. Modifications in the T-loop support the folding of the T-arm into its native state with the long-range tertiary contacts with the D-loop (25,29–31). The additional introduction of mnm^5^U balanced decoding of GGG and GGA codons by slightly impairing GGA but enhancing GGG. Uhlenbeck and colleagues have shown that the binding affinity of different tRNAs to their cognate codons in the ribosome is nearly uniform through a delicate interplay of amino acid identity, tRNA sequence and its modifications (80,81). Our results also indicate that the *E. coli* tRNA^Gly^ UCC provides the balanced and fine-tuned decoding of GGA and GGG codons only in presence of the full set of native modifications. The indispensability of T-arm modifications in conjunction with mature mnm^5^U, rather than precursor nm^5^U or m^5^U, highlights the sensitivity of decoding to even subtle chemical variations within modified nucleotides. This underscores the intricate and delicately balanced network of interactions governing this process. Also, in the superwobbling *M. mycoides* tRNA^Gly^ modifications contributed to the overall translation efficiency (Figure 5). While the s^4^U8 and P55 body modifications alone already increased the overall translation efficiency, they did not modulate the preference for specific codons. Only the full set including m^6^A37 slightly improved the decoding of GGU codons, resulting in a preference for A- and U-ending codons, which reflects the codon usage in *M. mycoides* (17).

Although our study on *M. mycoides* tRNA^Gly^ revealed the sequence as key prerequisite for efficient recognition of all four synonymous codons, it did not exclude a contribution of the modifications to superwobbling. Therefore, we aimed to confer the *E. coli* tRNA^Gly^ with superwobbling ability by the introduction of the *M. mycoides* tRNA anticodon loop modification. m^6^A at position 37 has previously been described as important for the preorganization (82), for limiting the flexibility (34) and for the prevention of a collapsed anticodon loop (29,83,84). Consequently, we hypothesized an increased binding affinity of the tRNA to the ribosomal A-site in presence of m^6^A37 to enable extended wobbling. Remarkably, m^6^A in the *E. coli* tRNA improved the its ability to decode all four synonymous codons (Figure 6). However, this beneficial effect of the m^6^A modification on four-way wobbling became apparent only when combined with the T-arm modifications, which might additionally support correct folding of the tRNA. The requirement for m^6^A37, T54 and P55 to arm the *E. coli* tRNA for efficient superwobbling again implies the importance of modification in stabilizing and prestructuring the entire tRNA molecule to achieve an optimized conformation for efficient participation in translation.

By employing chemically synthesized tRNAs a complete characterization of tRNA sequence and modifications on decoding can be performed. In the present study, the modifications of the *M. mycoides* tRNA^Gly^ were shown not to be essential for superwobbling. Instead, the tRNA sequence itself was identified as the primary prerequisite revealing the T-arm sequence as novel important element contributing to superwobbling. However, modifications modulate and improve decoding by being part of a delicate set of interactions required for efficient and accurate translation, possibly by facilitating the folding and prestructuring of the tRNA molecule. Our results highlight that the tRNA sequence and its modifications form a fully integrated system extremely complex to disentangle experimentally, requiring the need for novel approaches to study their impact on translation systematically.

## Supplementary Material

gkad1136_supplemental_file

## Data Availability

The data that support the findings of this study are available from the corresponding author upon request.
