# Supplementary material for: Contribution of tRNA sequence and modifications to the decoding preferences of E. coli and M. mycoides tRNAGlyUCC for synonymous glycine codons
Source: Nucleic Acids Res. 2023 Dec 5;52(3):1374–86. doi: 10.1093/nar/gkad1136 (PMC10853795; doi:10.1093/nar/gkad1136)
Supplement: gkad1136_supplemental_file [file gkad1136_supplemental_file.pdf]

SUPPLEMENTARY DATA

Contribution of tRNA sequence and modifications to the decoding preferences of *E. coli* and *M. mycoides* $tRNA^{Gly}$ UCC for synonymous glycine codons

Maria Kompatscher[1], Karolina Bartosik[2], Kevin Erharter[2], Raphael Plangger[2], Fabian Sebastian Juen[2], Christoph Kreutz[2], Ronald Micura[2], Eric Westhof[3], and Matthias Erlacher*[1]

[1]Institute of Genomics and RNomics, Biocenter, Medical University of Innsbruck, Innrain 80-82, 6020 Innsbruck, AUSTRIA
[2]Institute of Organic Chemistry, Leopold-Franzens University Innsbruck, Center for Chemistry and Biomedicine (CCB), Innrain 80-82, 6020 Innsbruck, AUSTRIA
[3]Architecture and Reactivity of RNA, Institute of Molecular and Cellular Biology of the CNRS UPR9002/University of Strasbourg, Strasbourg 67084, France

* To whom correspondence should be addressed. Tel: +43 512900370256; Fax: +43 512900373100; Email: matthias.erlacher@i-med.ac.at

SUPPLEMENTARY METHODS

In vitro **aminoacylation and acidic PAGE**

In vitro aminoacylation of 15 pmol tRNA was performed according to the protocol from (Ledoux and Uhlenbeck, 2008). tRNAs were aminoacylated employing recombinant GlyRS (NEB, P0822Z). The aminoacylation was performed at 37°C for 10 minutes and the reaction was stopped by adding 1x reaction volume of acidic sample buffer containing 0.1 M NaOAc (pH 5.2), 8 M urea, 0,05% bromophenol blue (adapted from Köhrer and RajBhandary, 2008). Samples were run on a 6.5% polyacrylamide (19:1), 8 M urea, 0.1 M NaOAc (pH 5.2) gel using 0.1 M NaoAC (pH 5.2) running buffer at 4°C for 20 hours at 100 V. Gel was stained with ethidium bromide and imaged by using Molecular Imager® Gel Doc™ XR Imaging System (Biorad).

Melting curve measurements of tRNAs

UV melting analysis

tRNA samples were dried in a concentrator and dissolved in 800 µl buffer (10 mM Na2HPO4, 150 mM NaCl, pH 7.0, containing 0 mM or 2.0 mM MgCl2), transferred into UV permeable high precision cells made of quartz SUPRASIL® with a light path of 10 mm and layered with polydimethylsiloxane to avoid evaporation. UV melting profiles were recorded at 250 and 260 nm on an Agilent Cary 3500 UV-Vis spectrophotometer equipped with a multiple cell holder and a Peltier temperature control device. Each tRNA was measured at the 0.5 µM concentration and with at least four ramps (heating-cooling-heating-cooling; 0.7 °C min–1 heating/cooling rate; temperature scope from 5 °C to 90 °C). Melting profiles were determined according to the absorbance data from the third ramp (cooling).

NMR melting analysis

Enzymatic RNA synthesis: For *in vitro* transcription of each tRNA studied by NMR spectroscopy, 0.0011 mM DNA template and 0.0010 mM T7 promotor strand were annealed at 90°C. A tenth of the desired reaction volume 10x transcription buffer (1 M Tris-glutamate, 20 mM spermidine, pH 8.2), 20 mM DTT, 4 mM of each rNTP, 25 mM $MgCl_2$, 10% (v/v)

dimethylsulfoxide and 0.15 mM T7 RNA polymerase (expressed in-house) were added and incubated overnight at 37°C. The precipitated magnesium salts were dissolved by addition of 1.5 eq. EDTA (0.5 M, pH 8.0) in relation to the Mg^{2+} concentration. A tenth of the volume of a sodium acetate solution (3 M, pH 5.6) was added to adjust the pH of the solution. The crude RNA was sterile filtered (0.22 µm) and directly applied to an inhouse packed desalting column (Interchim puriflash L x I.D. 150 mm x 60 mm, Sephadex™ G-25 Fine, cytiva, Austria) using a ÄKTA start system (GE Healthcare, Austria). It was eluted using HPLC grade water and the RNA containing fractions (UV detection at 254 nm) were collected in a 100 ml round bottom flask. After evaporation, the crude RNA was dissolved in 1 ml HPLC grade water and transferred to a 1.5 ml reaction tube. The crude RNA was stored at -20°C. The quality of the crude RNAs was checked via anion exchange chromatography on an analytical Dionex DNAPac PA-200 column (4x250 mm; Eluent A: 25 mM Tris-HCl, 10 mM sodium perchlorate, 20 % acetonitrile, pH 8.0; Eluent B: 25 mM Tris-HCl, 600 mM sodium perchlorate, 20 % acetonitrile, pH 8.0) and at elevated temperature (80 °C). Purification of the RNA sequences was achieved in a single run by applying the crude RNA to a preparative Dionex DNAPac PA-200 column (22x250 mm, eluents as before). The fractions containing the desired RNA were pooled and loaded on a C18 SepPak catridge (Waters, Austria) to remove HPLC buffer salts. The RNA sodium salt form was then eluted from the C18 column with water/acetonitrile (1/1, v/v), concentrated and transferred to a 1.5 ml reaction tube for concentration determination and mass spectrometric analysis. Sample concentrations were determined by measuring UV absorption at 260 nm on a NanoPhotometer (Implen).

LC-ESI mass spectrometry: All RNAs were analyzed on Finnigan LCQ Advantage MAX ion trap instrumentation connected to a Thermo Scientific UHPLC (components: Ultimate 3000 RS Pump, Ultimate 3000 RS Autosampler, Ultimate 3000 RS Column Compartment, Ultimate 3000 Diode Array Detector). RNA mass spectra were acquired in the negative-ion mode with a potential of -4 kV applied to the spray needle (capillary temperature: 270°C, capillary voltage: -23V). LC: 250 pmol RNA dissolved in 30 µl of 20 mM ethylenediaminetetraacetic acid (EDTA) solution; average injection volume: 30 µl; column: Waters xBridge C18 2.5µm column (1.0 × 50 mm) at 30°C; flow rate: 100 µl/min; Eluent A: 8.6 mM triethylamine (TEA), 100 mM 1,1,1,3,3,3-hexafluoroisopropanol in H2O (pH 8.0); Eluent B: methanol; gradient: 0–100% B in A within 30 min; UV detection at 260/280 nm. The correct assembly of all RNAs used in this study was confirmed by the mass data.

NMR spectroscopy: RNA samples were lyophilized as sodium salts and dissolved in 450 µL NMR buffer (15 mM sodium phosphate, 25 mM NaCl, 0.1% NaN_3, pH 6.5, 10 % D_2O) and transferred into standard 5 mm NMR tubes giving sample concentrations between 111 µM and 278 µM. Correct folding was induced by a heat shock snap cooling procedure by heating the RNA to 90 °C for 2 minutes followed by rapid cooling in an ice water bath and addition of MgCl2 to a final concentration of 10 mM.

Experiments were run at 288 K, 298 K, 308 K, 318 K, 328 K, 333 K, 338 K, 434 K, 348 K, 353 K, 358 K. All NMR experiments were acquired on a Bruker 700 MHz Avance Neo NMR equipped with a Prodigy TCI probe.

All datasets were processed with Topspin 4.2.0 (Bruker Biospin).

SUPPLEMENTARY FIGURES

```
E. coli
GCGGGAAUAGCUCAGUUGGU.AGAGCACGACCUUGCCAAGGUCGGG..........GUCGCGAGUUCGAGUCUCGUUUCCCGCUCCA 4 Sc: 88.4
GCGGGAA4AGCUCAGDDGGD.AGAGCACGACCUUGCCAAGGUCGGG..........7UCGCGAGTPCGAGUCUCGUUUCCCGCUCCA
GCGGGCGUAGUUCAAU.GGU.AGAACGAGAGCUUCCCAAGCUCUAU..........A.CGAGGGUUCGAUUCCCUUCGCCCGCUCCA 1 Sc: 81.1
GCGGGCG4AGUUCAAU.GGD.AGAACGAGAGCUUCCCAAGCUCUAU..........A.CGAGGGTPCGAUUCCCUUCGCCCGCUCCA
GCGGGCAUCGUAUAAU.GGCUAUUACCUCAGCCUUCCAAGCUGAUG..........A.UGCGGGUUCGAUUCCCGCUGCCCGCUCCA 1 Sc: 70.6
GCGGGCAUCGUAUAAU.GGCUAUUACCUCAGCCU{CCAAGCUGAUG..........A.UGCGGGTPCGAUUCCCGCUGCCCGCUCCA

Micrococcus luteus (high G/C content > 75%)
GCGGAUGUAGCUCAGUUGGCUAGAGCGCCACCUUGCCAAGGUGGAG..........GUCGCGAGUUCGAAUCUCGUCAUCCGCUCCA 1 Sc: 91.6
GCGGAUGUAGCUCAGUUGGCUAGAGCGCCACCUUGCCAAGGUGGAG..........GUCGCGAGUUCGAAUCUCGUCAUCCGCU... 1 Sc: 84.8
GCGGGCGUAGCUCAAU.GGU.AGAGCGCUUGCUUCCCAAGCAAGAU..........A.CGCGGGUUCGAUUCCCGUCGCCCGCUCCA 1 Sc: 88.6
GGGGAUGUAGCUCAAU.GGU.AGAGCCCAGUCUUCCAAACUGGCU..........A.CGCGGGUUCGAUUCCCGUCAUCCCCU... 1 Sc: 78.2

M. pneumoniae (intermediate G/C content = 40%)
GCAGAUAUAGUUCAAU.GGC.AGAACAUAACCUUGCCAAGGUUAAG..........A.UGCGGGUUCGAUUCCCGUUAUCUGCUCCA 1 Sc: 78.9
GCGAGUAUAGUUUAGU.GGU.AGAACAUCAGCCUUCCAAGCUGAUC..........G.UGUCGGUUCGAUUCCGAUUACUCGCUCCA 1 Sc: 74.9

Mycoplasma genitalium (low G/C content > 25%)
GCAGAUAUAGUUCAAU.GGU.AGAACAUAACCUUGCCAAGGUUAAG..........A.UGCGGGUUCGAUUCCCGUUAUCUGCUCCA 1 Sc: 80.2
GCGAGUAUAGUUUAAU.GGU.AGAACAUCAGCCUUCCAAGCUGAUC..........G.UGUCGGUUCGAUUCCGAUUACUCGCUCCA 1 Sc: 73.6

M. mycoides (lowest G/C content = 24%)
GCAGGUGUAGUUUAAU.GGC.AGAACUUCAGCCUUCCAAGCUGAUU..........G.UGAGGGUUCGAUUCCCUUCACCUGCUCCA 1 Sc: 75.3
GCAGGUG4AGUUUAAU.GGC.AGAACUUCAGCCUUCC=AGCUGAUU..........G.UGAGGGTPCGAUUCCCUUCACCUGCUCCA

M. capricolum (lowest G/C content = 24%)
GCAGGUGUAGUUUAAU.GGU.AGAACUUCAGCCUUCCAAGCUGAUU..........G.UGAGGGUUCGAUUCCCUUCACCUGCUCCA 1 Sc: 76.6
GCAGGUG4AGUUUAAU.GGD.AGAACUUCAGCCUUCC=AGCUGAUU..........G.UGAGGGUPCGAUUCCCUUCACCUGCUCCA
```

Figure S1. Alignment of $tRNA^{Gly}$ sequences of bacteria with different genomic GC content. tRNA sequences are provided without modifications extracted from GtRNAdb (Chan and Lowe, 2016) and duplicated with tRNA modifications available in MODOMICS (Boccaletto et al., 2022). 4…s4U, 7…m7G, D…dihydrouridine, P…pseudouridine, T…5-methyluridine, {…5-methylaminomethyluridine, =…N6-methyladenosine). The colour scheme indicates AA-stem (yellow), D-arm (green), AC-arm (cyan), T-arm (magenta). The scores at the right are those from GtRNAdb. In the *E. coli* $tRNA^{Gly}$ sequences, some key variations with respect to the other species are shown in red (positions 9, 12, 13, 22, 23).

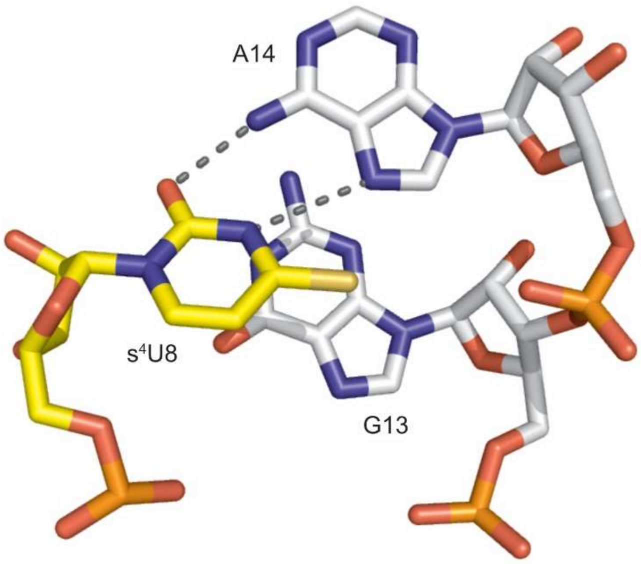

Figure S2. The role of 4-thiouridine (s^4U) at tRNA position 8 in stacking and stabilization. The 4-thio group of the s^4U stacks with the nucleotide at position 13 and base pairs via W/C or Hoogsteen base pairing with nucleotide at position 14. The depicted structure was extracted from $tRNA^{Tyr}$ within the P-site of a 70S ribosome (4WZD) (Rozov et al., 2015) using PyMOL.

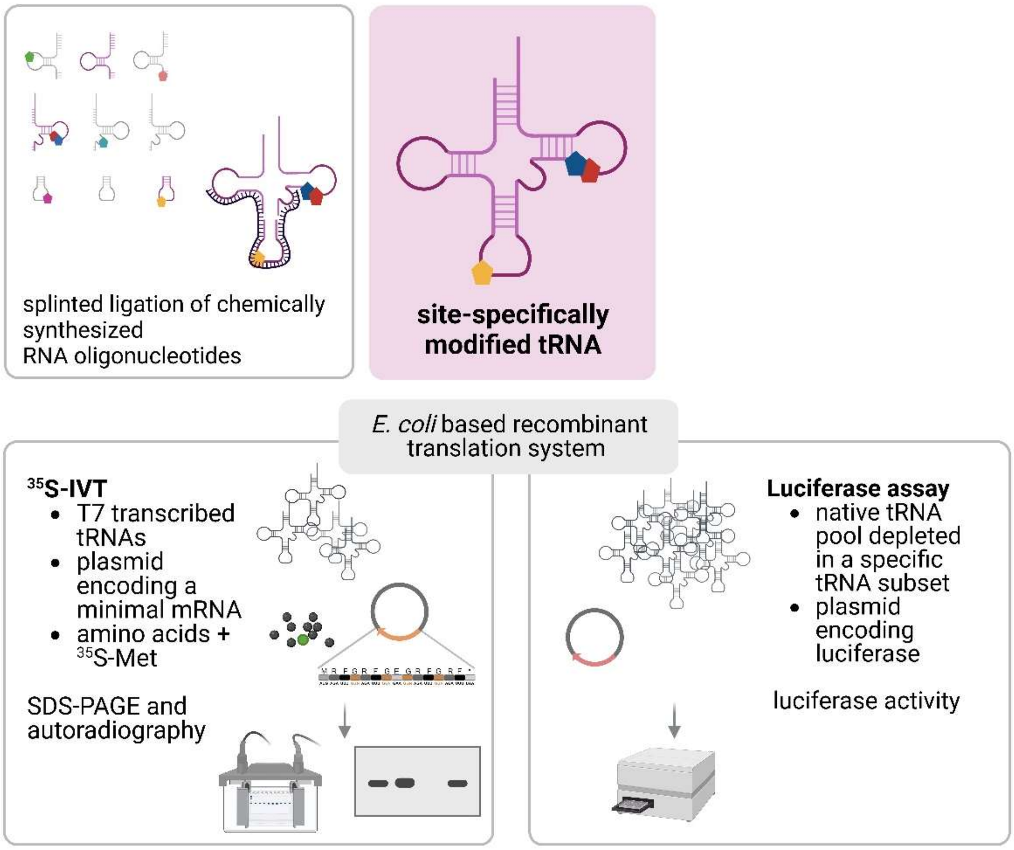

Figure S3. Splinted ligation of tRNAs and *in vitro* translation assay. Upper panels show differently modified RNA oligonucleotides, which are ligated with the aid of a DNA splinter and T4 DNA ligase to generate site-specifically modified tRNAs. Lower left panel depicts the *in vitro* translation assay in presence of [^{35}S]-methionine to translate a small mRNA encoded on a plasmid into an approximately 1.7 kDa peptide. Lower right panel shows the *in vitro* translation assay of a firefly luciferase gene by the use of tRNA^{Gly} depleted *E. coli* tRNA bulk.

fLuc coding sequence (550 codons + stop codon):

ATG GAA GAC GCC AAA AAC ATA AAG AAA **GGC** CCG GCG CCA TTC TAT CCG CTG GAA GAT **GGA** ACC GCT **GGA** GAG CAA CTG CAT AAG GCT ATG AAG AGA TAC GCC CTG GTT CCT **GGA** ACA ATT GCT TTT ACA GAT GCA CAT ATC GAG GTG GAC ATC ACT TAC GCT GAG TAC TTC GAA ATG TCC GTT CGG TTG GCA GAA GCT ATG AAA CGA TAT **GGG** CTG AAT ACA AAT CAC AGA ATC GTC GTA TGC AGT GAA AAC TCT CTT CAA TTC TTT ATG CCG GTG TTG **GGC** GCG TTA TTT ATC **GGA** GTT GCA GTT GCG CCC GCG AAC GAC ATT TAT AAT GAA CGT GAA TTG CTC AAC AGT ATG **GGC** ATT TCG CAG CCT ACC GTG GTG TTC GTT TCC AAA AAG **GGG** TTG CAA AAA ATT TTG AAC GTG CAA AAA AAG CTC CCA ATC ATC CAA AAA ATT ATT ATC ATG GAT TCT AAA ACG GAT TAC CAG **GGA** TTT CAG TCG ATG TAC ACG TTC GTC ACA TCT CAT CTA CCT CCC **GGT** TTT AAT GAA TAC GAT TTT GTG CCA GAG TCC TTC GAT AGG GAC AAG ACA ATT GCA CTG ATC ATG AAC TCC TCT **GGA** TCT ACT **GGT** CTG CCT AAA **GGT** GTC GCT CTG CCT CAT AGA ACT GCC TGC GTG AGA TTC TCG CAT GCC AGA GAT CCT ATT TTT **GGC** AAT CAA ATC ATT CCG GAT ACT GCG ATT TTA AGT GTT GTT CCA TTC CAT CAC **GGT** TTT **GGA** ATG TTT ACT ACA CTC **GGA** TAT TTG ATA TGT **GGA** TTT CGA GTC GTC TTA ATG TAT AGA TTT GAA GAA GAG CTG TTT CTG AGG AGC CTT CAG GAT TAC AAG ATT CAA AGT GCG CTG CTG GTG CCA ACC CTA TTC TCC TTC TTC GCC AAA AGC ACT CTG ATT GAC AAA TAC GAT TTA TCT AAT TTA CAC GAA ATT GCT TCT **GGT** **GGC** GCT CCC CTC TCT AAG GAA GTC **GGG** GAA GCG GTT GCC AAG AGG TTC CAT CTG CCA **GGT** ATC AGG CAA **GGA** TAT **GGG** CTC ACT GAG ACT ACA TCA GCT ATT CTG ATT ACA CCC GAG GGG GAT GAT AAA CCG **GGC** GCG GTC **GGT** AAA GTT GTT CCA TTT TTT GAA GCG AAG GTT GTG GAT CTG GAT ACC GGG AAA ACG CTG **GGC** GTT AAT CAA AGA **GGC** GAA CTG TGT GTG AGA **GGT** CCT ATG ATT ATG TCC **GGT** TAT GTA AAC AAT CCG GAA GCG ACC AAC GCC TTG ATT GAC AAG GAT **GGA** TGG CTA CAT TCT **GGA** GAC ATA GCT TAC TGG GAC GAA GAC GAA CAC TTC TTC ATC GTT GAC CGC CTG AAG TCT CTG ATT AAG TAC AAA **GGC** TAT CAG GTG GCT CCC GCT GAA TTG GAA TCC ATC TTG CTC CAA CAC CCC AAC ATC TTC GAC GCA **GGT** GTC GCA **GGT** CTT CCC GAC GAT GAC GCC **GGT** GAA CTT CCC GCC GCC GTT GTT GTT TTG GAG CAC **GGA** AAG ACG ATG ACG GAA AAA GAG ATC GTG GAT TAC GTC GCC AGT CAA GTA ACA ACC GCG AAA AAG TTG CGC **GGA** **GGA** GTT GTG TTT GTG GAC GAA GTA CCG AAA **GGT** CTT ACC **GGA** AAA CTC GAC GCA AGA AAA ATC AGA GAG ATC CTC ATA AAG GCC AAG AAG **GGC** **GGA** AAG ATC GCC GTG TAA

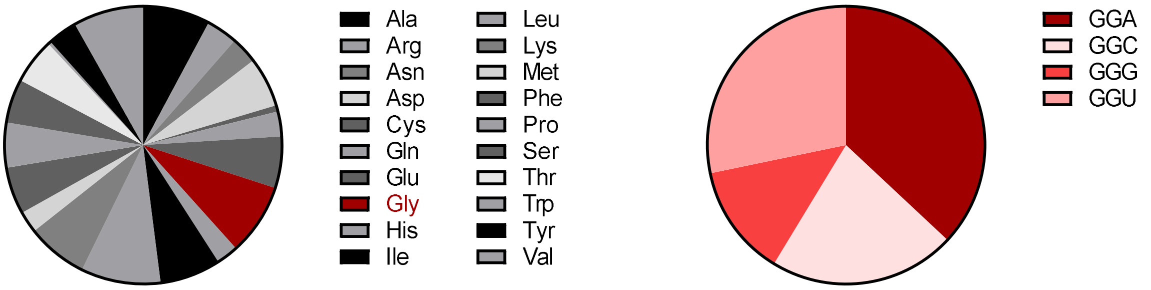

Total=550 **Total=46**

Figure S4. Firefly luciferase (fLuc) coding sequence (CDS) consists in 551 codons including the stop codon. Amino acid composition of the fLuc protein is shown (glycine content highlighted in red). The CDS encodes for 46 glycine codons (17 GGA, 10 GGC, 7 GGG, and 13 GGU codons).

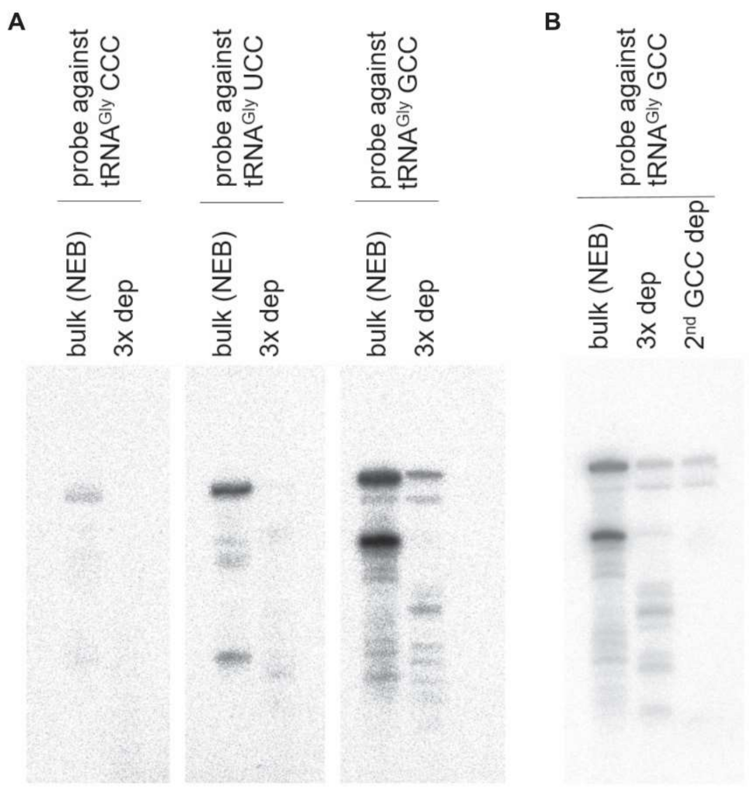

Figure S5. Depletion of tRNA^{Gly} isoacceptors in *E. coli* tRNA bulk by RNase H digestion. (**A**) Northern blots were performed to determine the efficiency of depleting *E. coli* tRNA^{Gly} isoacceptors. (**B**) Due to the abundance of the *E. coli* tRNA^{Gly} GCC, the tRNA was depleted a second time. Bulk…untreated tRNA pool, 3x dep…simultaneous digestion of the three isoacceptors by RNase H, 2nd GCC dep… additional RNase H digestion of the tRNA^{Gly} GCC;

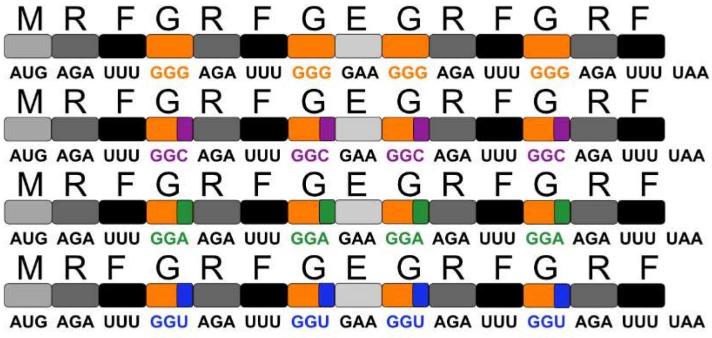

Figure S6. Sequences of short mRNAs encoding for an approximately 1.7 kDa peptide encoded by a small set of codons. The mRNAs only differ in the third nucleotide of the glycine codons to be able to determine the decoding capability of the tested tRNAs.

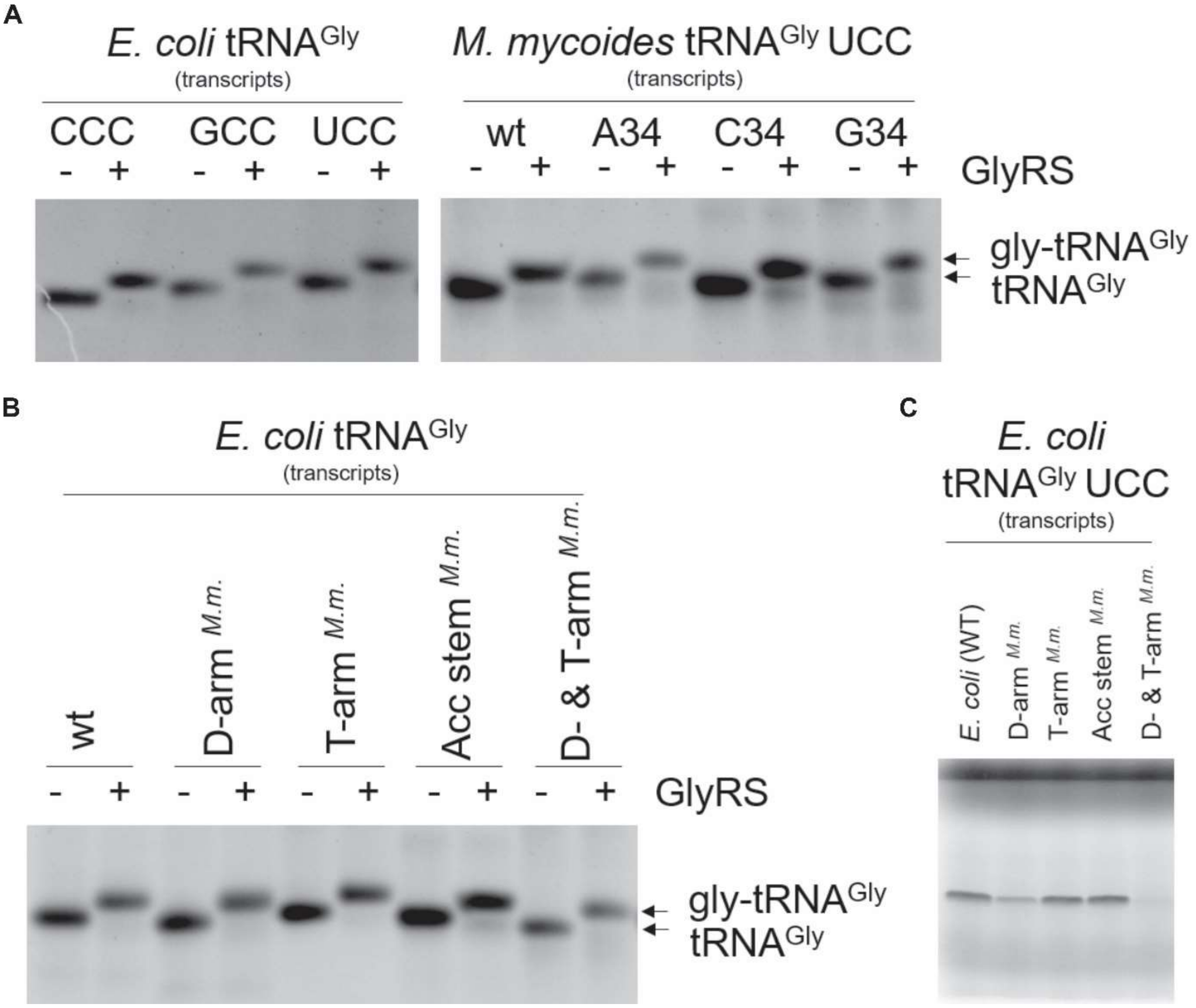

Figure S7. Aminoacylation of tRNA variants by the *E. coli* glycyl-tRNA synthetase (GlyRS). Acidic PAGE of *in vitro* aminoaclylated tRNA transcripts: (**A**) *E. coli* $tRNA^{Gly}$ isoacceptors, *M. mycoides* $tRNA^{Gly}$ UCC wild type and mutants, and (**B**) *E. coli* $tRNA^{Gly}$ UCC mutants. (**C**) Autoradiograph subsequent *in vitro* translation of short mRNA encoding GGA codons by the *in vitro* transcribed *E. coli* $tRNA^{Gly}$ UCC mutants.

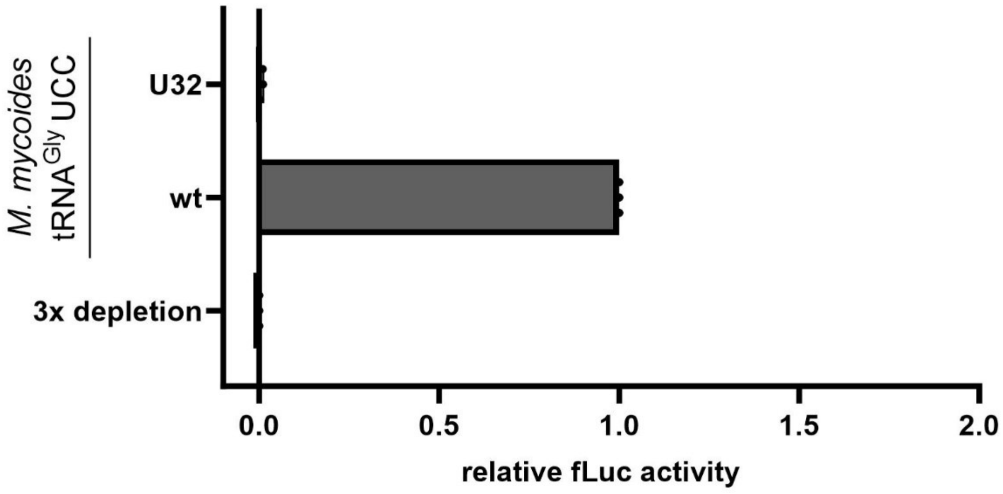

Figure S8. Impact of the C32U mutation on superwobbling. Mutant and wild type (wt) *M. mycoides* $tRNA^{Gly}$ UCC were compared in their ability to sustain fLuc translation in absence of the native *E. coli* tRNA isoacceptors (3x depletion). Datapoints represent technical duplicates of individual experiments. The fLuc activity was normalized to the wt *M. mycoides* tRNA sample.

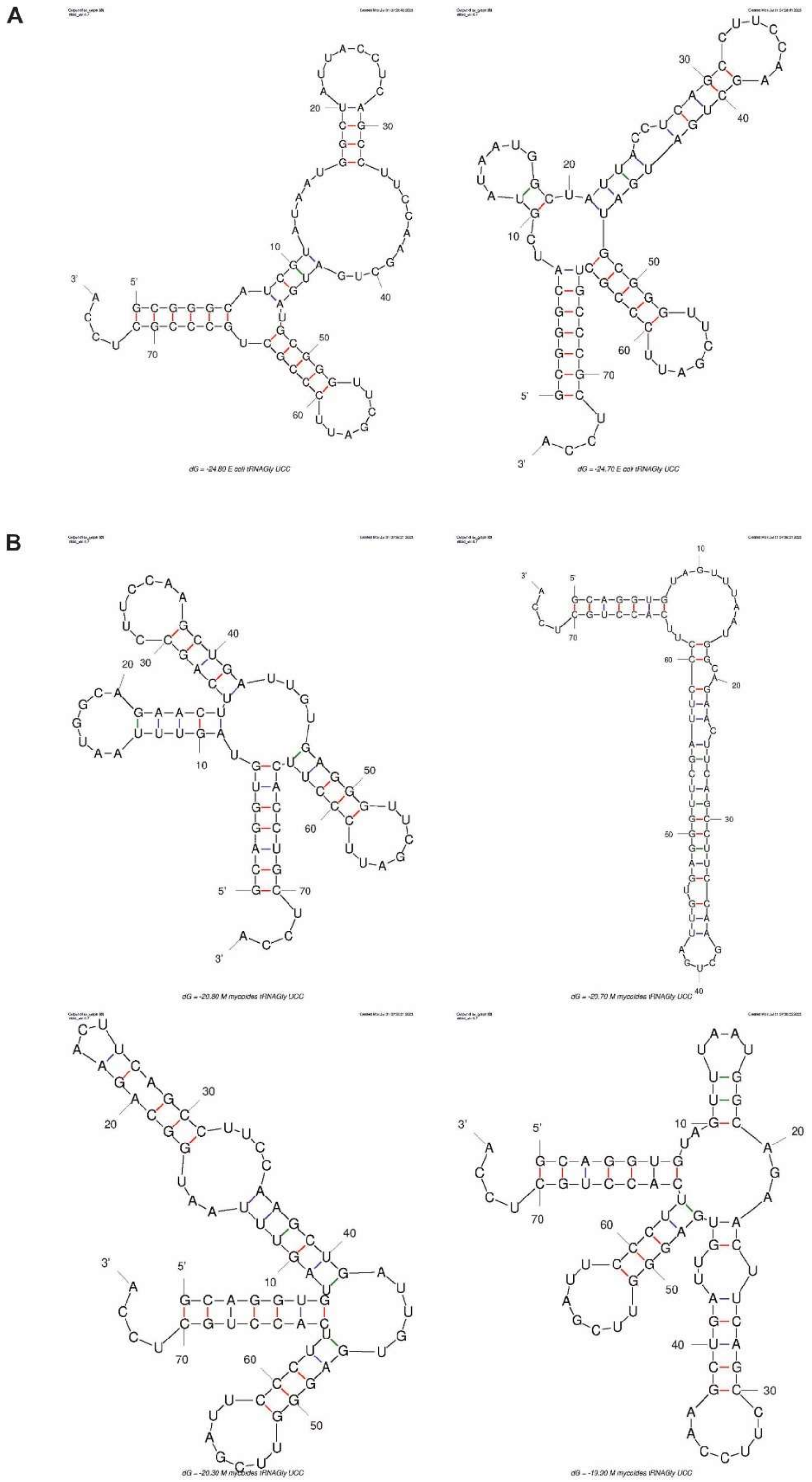

Figure S9. Secondary structure prediction of tRNA sequences by mfold version 2.3 (Zuker, 2003). (**A**) Structural prediction of *E. coli* $tRNA^{Gly}$ UCC. In the structure at the right, the AA- and T-stems are correctly predicted but not the other two stems. (**B**) Structural prediction of *M. mycoides* $tRNA^{Gly}$ UCC. The first predicted fold presents all stems correctly except for the additional base pair at the beginning of the D-stem. Except for one, all the other predictions have the correct AA- and T-stems. The predicted structures provided by the software are shown in decreasing ΔG values.

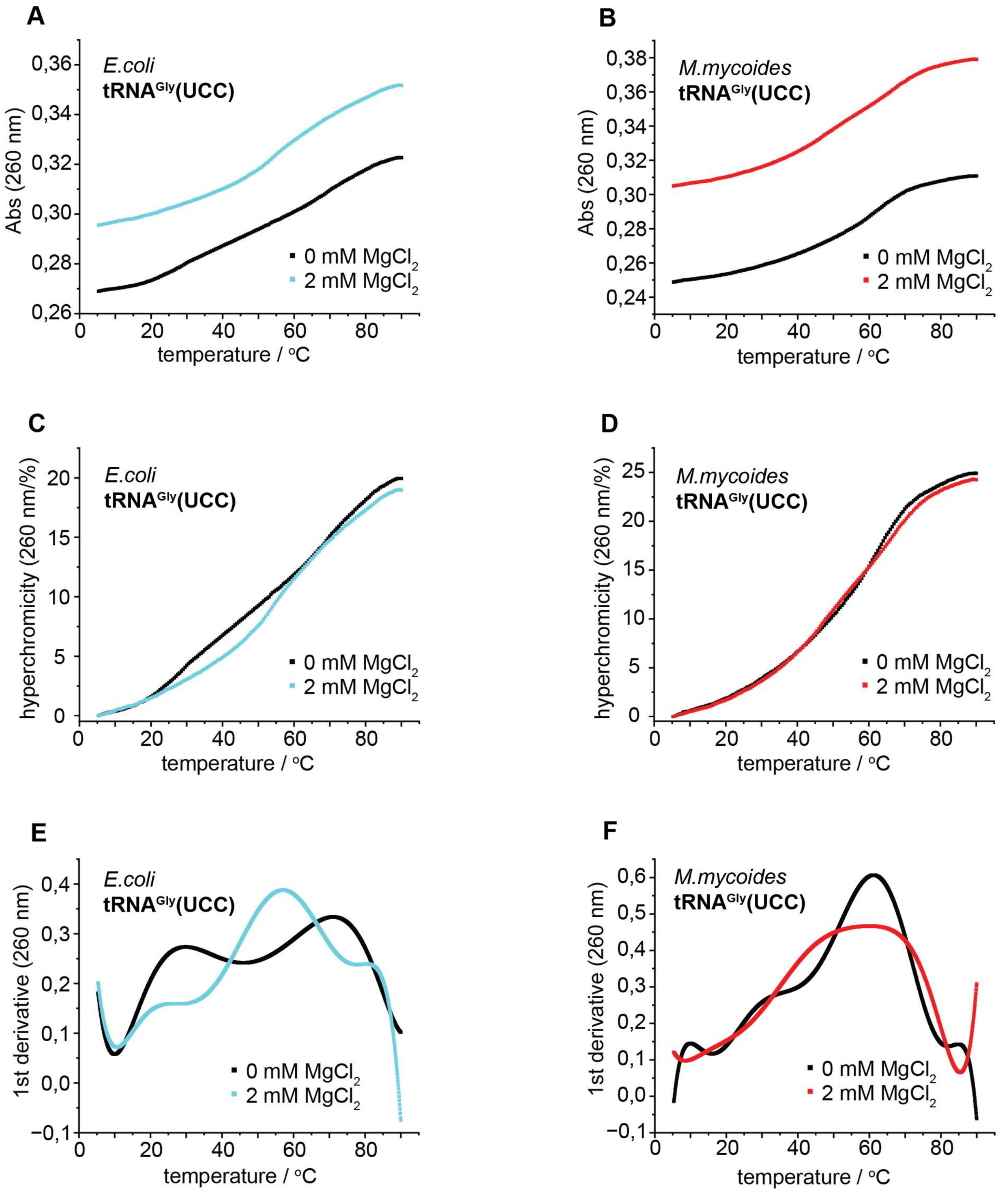

Figure S10. UV melting profile analyses of (A, C, E) *E. coli* tRNA^{Gly}(UCC) and (B, D, F) *M. mycoides* tRNA^{Gly}(UCC) in the absence and in the presence of Mg^{2+} ions. For each tRNA an absorbance (top), hyperchromicity (middle) and its derivative (bottom) were plotted as a function of temperature. The plots indicate that the presence of 2 mM $MgCl_2$ increase the folded states of the tRNAs. The plots E and F show a difference between *E. coli* and *M. mycoides* tRNAs In absence of $MgCl_2$, the *E. coli* tRNA has at least two states that merge together in presence of $MgCl_2$; while the *M. mycoides* tRNA presents a broad peak in absence of $MgCl_2$ that sharpen in presence of $MgCl_2$.

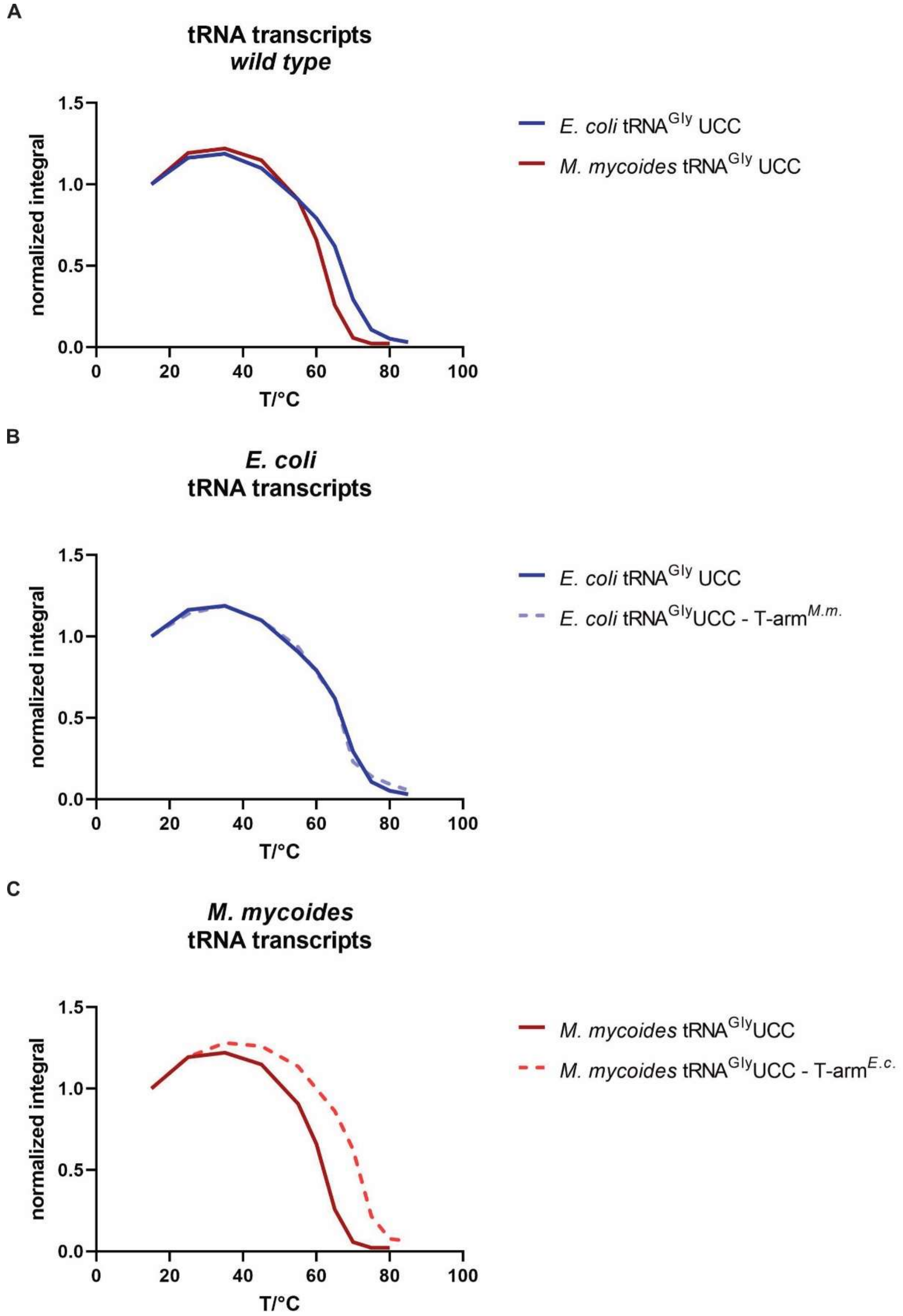

Figure S11. NMR melting analysis of unmodified tRNA^{Gly} UCC transcripts in presence of 10 mM Mg^{2+} ions. (**A**) Normalized integral of the NMR imino spectrum of *E. coli* (blue) and *M. mycoides* (red) tRNA^{Gly} UCC wild type sequences. (**B**) *E. coli* and (**C**) *M. mycoides* wild type tRNA sequence (solid line) and tRNA with exchanged T-arm (dashed line) were compared.

Supplementary Table S1. tRNA gene sequences were obtained from tRNA Leipzig Database (Jühling et al., 2009). Primer for PCR amplification are provided with their annealing temperature.

tRNA template	organism	primer sequence	annealing temp.	Database ID	tRNA gene sequence
$tRNA^{Arg}$ UCU	*E. coli*	*GGATCCTAATACGACTCACTATA* GCGCCCTTAGCT**CAGTTGGATAGAGCAACG**<br>mUmGGCGCGCCCTGCAGGATTCGAACCTGCGGCCCACGACTTAGAAGGT**CGTTGCTCTATCCAACTG**	59°C	tdbD00002451	GCGCCCTTAGCTCAGTTGGATAGAGCAACGACCTTCTAAGTCGTGGCCGCAGGTTCGAATCCTGCAGGGCGCGCCA
$tRNA^{Gly}$ CCC	*E. coli*	*GGATCCTAATACGACTCACTATA* GCGGGCGTAGTTCAATGGTAGAA**CGAGAGCTTCCCAAGCTCTATACG**<br>mUmGGAGCGGGCGAAGGGAATCGAACCCT**CGTATAGAGCTTGGGAAGCTCTCG**	67°C	tdbD00000839	GCGGGCGTAGTTCAATGGTAGAACGAGAGCTTCCCAAGCTCTATACGAGGGTTCGATTCCCTTCGCCCGCTCCA
$tRNA^{Gly}$ GCC	*E. coli*	*GGATCCTAATACGACTCACTATA* GCGGGAATAGCTCA**GTTGGTAGAGCACGACC**<br>mUmGGAGCGGGAAACGAGACTCGAACTCGCGACCCCGACCTTGGCAA***GGTCGTGCTCTACCAAC***	60°C	tdbD00000838	GCGGGAATAGCTCAGTTGGTAGAGCACGACCTTGCCAAGGTCGGGGTCGCGAGTTCGAGTCTCGTTTCCCGCTCCA

tRNA^{Gly} UCC	*E. coli*	*GGATCCTAATACGACTCACTATA* GCGGGCATC**GTATAATGGCTATTACCTCAGC**<br>mUmGGAGCGGGCAGCGGGAATCGAACCCGCATCATCAGCTT**GGA**AG**GCTGAGGTAATAGCCATTATAC**	59°C	tdbD00000837	GCGGGCATCGTATAATGGCTATTACCTCAGCCTTCCAAGCTGATGATGCGGGTTCGATTCCCGCTGCCCGCTCCA
tRNA^{fMet} CAU	*E. coli*	*GGATCCTAATACGACTCACTATA* CGCGGGGTGGAGC**AGCCTGGTAGCTCGTCG**<br>mUmGGTTGCGGGGGCCGGATTTGAACCGACGACCTTCGGGTTATGAGCC**CGACGAGCTACCAGGCT**	63°C	tdbD00003430	CGCGGGGTGGAGCAGCCTGGTAGCTCGTCGGGCT*CAT*AACCCGAAGGTCGTCGGTTCAAATCCGGCCCCCGCAACCA
tRNA^{Phe} GAA	*E. coli*	*TTCTAATACGACTCACTATA* GCCCGGATAGCTCAGTCGG**TAGAGCAGGGGATTGAAA**<br>TGGTGCCCGGACTCGGAATCGAACCAAGGACACGGGGAT**TTTCAATCCCCTGCTCTA**	59°C	tdbD00000686	GCCCGGATAGCTCAGTCGGTAGAGCAGGGGATTGAAAATCCCCGTGTCCTTGGTTCGATTCCGAGTCCGGGCACCA
tRNA^{Gly} UCC	*M. mycoides*	*GGATCCTAATACGACTCACTATA* GCAGGTGTAGTTTAATGGCAGAA**CTTCAGCCTTCCAAGCTGATT**<br>mUmGGAGCAGGTGAAGGGAATCGAACCCTCAC**AATCAGCTTGGAAGGCTGAAG**	63°C	tdbD00000812	GCAGGTGTAGTTTAATGGCAGAACTTCAGCCTTCCAAGCTGATTGTGAGGGTTCGATTCCCTTCACCTGCTCCA

Supplementary Table S2. Sequences of antisense DNA oligonucleotides used for RNase H digestion and splinted ligation.

tRNA target	DNA sequence (5'-3')
E. coli tRNA^{Gly} CCC	TGGAGCGGGCGAAGGGAATCGAACCCTCGTATAGAGCTTGGGAAGCTCTCGTTCTAC
E. coli tRNA^{Gly} GCC	ACTCGCGACCCCGACCTTGGCAAGGTCGTGCTCTACCAACTGA
E. coli tRNA^{Gly} UCC	AACCCGCATCATCAGCTTGGAAGGCTGAGGTAATAGCCATTAT
M. mycoides tRNA^{Gly} UCC	AACCCTCACAATCAGCTTGGAAGGCTGAAGTTCTGCCATTAAA

Supplementary Table S3. Sequences of the RNA oligonucleotides harbouring the studied RNA modifications.

**tRNA**	sequence (5'-3')	modifi-cation	obtained from
E. coli tRNA^{Gly} UCC	GCGGGCAUCGUAUAAUGGCUAUUACC	-	Micura lab / IDT
	UCAGCCUUCCAAG	-	Micura lab / IDT
	UCAGCCU<m5U>CCAAG	m5U	IDT
	GCGGGCAUCGUAUAAUGGCUAUUACCUCAGCCU<nm5U>CCAAG	nm5U	Kreutz lab
	GCGGGCAUCGUAUAAUGGCUAUUACCUCAGCCU<mnm5U>CCAAG	mnm5U	Kreutz lab
	CUGAUGAUGCGGGUUCGAUUCCCGCUGCCCGCUCCA	-	Micura lab / IDT
	CUGAUGAUGCGGG<T><P>CGAUUCCCGCUGCCCGCUCCA	T, P	Kreutz lab
M. mycoides tRNA^{Gly} UCC	GCAGGUGUAGUUUAAUGGCAGAACU	-	IDT
	GCAGGUG<s4U>AGUUUAAUGGCAGAACU	s4U	Micura lab
	UCAGCCUUCCAAG	-	IDT
	UCAGCCUUCC<m6A>AG	m6A	Dharmacon
	CUGAUUGUGAGGGUUCGAUUCCCUUCACCUGCUCCA	-	IDT
	CUGAUUGUGAGGGU<P>CGAUUCCCUUCACCUGCUCCA	P	Dharmacon

References

Boccaletto, P., Stefaniak, F., Ray, A., Cappannini, A., Mukherjee, S., Purta, E., et al. (2022). MODOMICS: A database of RNA modification pathways. 2021 update. *Nucleic Acids Res.* 50, D231–D235. doi: 10.1093/nar/gkab1083.

Chan, P. P., and Lowe, T. M. (2016). GtRNAdb 2.0: An expanded database of transfer RNA genes identified in complete and draft genomes. *Nucleic Acids Res.* 44, D184–D189. doi: 10.1093/nar/gkv1309.

Rozov, A., Demeshkina, N., Westhof, E., Yusupov, M., and Yusupova, G. (2015). Structural insights into the translational infidelity mechanism. *Nat. Commun.* 6. doi: 10.1038/ncomms8251.

Zuker, M. (2003). Mfold web server for nucleic acid folding and hybridization prediction. *Nucleic Acids Res.* 31, 3406–3415. doi: 10.1093/nar/gkg595.
